# MIL-125-based nanocarrier decorated with Palladium complex for targeted drug delivery

**DOI:** 10.1038/s41598-022-16058-w

**Published:** 2022-07-15

**Authors:** Mojtaba Bagherzadeh, Moein Safarkhani, Mahsa Kiani, Fatemeh Radmanesh, Hossein Daneshgar, Amir Mohammad Ghadiri, Fahimeh Taghavimandi, Yousef Fatahi, Nahid Safari-Alighiarloo, Sepideh Ahmadi, Navid Rabiee

**Affiliations:** 1 Department of Chemistry, Sharif University of Technology, Tehran, Iran; 2 Uro-Oncology Research Center, Tehran University of Medical Sciences, Tehran, Iran; 3 Department of Stem Cells and Developmental Biology, Cell Science Research Center, Royan Institute for Stem Cell Biology and Technology, ACECR, Tehran, Iran; 4 Nanotechnology Research Centre, Faculty of Pharmacy, Tehran University of Medical Sciences, Tehran, Iran; 5 Department of Pharmaceutical Nanotechnology, Faculty of Pharmacy, Tehran University of Medical Sciences, Tehran, Iran; 6 Universal Scientific Education and Research Network (USERN), Tehran, Iran; 7 Endocrine Research Center, Institute of Endocrinology and Metabolism, Iran University of Medical Sciences, Tehran, Iran; 8 Department of Medical Biotechnology, School of Advanced Technologies in Medicine, Shahid Beheshti University of Medical Sciences, Tehran, Iran; 9 School of Engineering, Macquarie University, Sydney, NSW 2109 Australia; 10 Department of Materials Science and Engineering, Pohang University of Science and Technology (POSTECH), 77 Cheongam-ro, Nam-gu, Pohang, Gyeongbuk 37673 South Korea

**Keywords:** Biochemistry, Drug discovery

## Abstract

The aim of this work was to provide a novel approach to designing and synthesizing a nanocomposite with significant biocompatibility, biodegradability, and stability in biological microenvironments. Hence, the porous ultra-low-density materials, metal–organic frameworks (MOFs), have been considered and the MIL-125(Ti) has been chosen due to its distinctive characteristics such as great biocompatibility and good biodegradability immobilized on the surface of the reduced graphene oxide (rGO). Based on the results, the presence of transition metal complexes next to the drug not only can reinforce the stability of the drug on the structure by preparing π–π interaction between ligands and the drug but also can enhance the efficiency of the drug by preventing the spontaneous release. The effect of utilizing transition metal complex beside drug (Doxorubicin (DOX)) on the drug loading, drug release, and antibacterial activity of prepared nanocomposites on the *P. aeruginosa* and *S. aureus* as a model bacterium has been investigated and the results revealed that this theory leads to increasing about 200% in antibacterial activity. In addition, uptake, the release of the drug, and relative cell viabilities (in vitro and in vivo) of prepared nanomaterials and biomaterials have been discussed. Based on collected data, the median size of prepared nanocomposites was 156.2 nm, and their biological stability in PBS and DMEM + 10% FBS was screened and revealed that after 2.880 min, the nanocomposite’s size reached 242.3 and 516 nm respectively. The MTT results demonstrated that immobilizing PdL beside DOX leads to an increase of more than 15% in the cell viability. It is noticeable that the AST:ALT result of prepared nanocomposite was under 1.5.

## Introduction

Drug delivery systems are considered one of the most remarkable approaches in human health care and this field is ever-evolving in biomedical science^1–4^. For delivering compounds to the targeted cells, knowing the features of tumor cells is momentous. The tumor cells have some unique characteristics like lower pH, hypoxia, redox potential, abnormal vascular, overexpression, and spatial texture^5–8^. The pH of tumor cells is about one unit less than normal cells and regarding to the tumor type, it could be in the range of 5.5—6.7. The tumor cells need more oxygen and nutrients, so they create atypical vessels, and as a result of more activity and metabolism, they lack oxygen^9–12^. As a consequence of being gluttonous, the mutated cells express their on-the-cell receptors more than normal cells. These features helped scientists develop a more optimized carrier to enhance the loading and improve the drug release. The carrier has some characteristics, and the most important of them is the size^13–15^. If the particle size is < 40 nm, the penetration rate will be so fast because it can easily pass through the endothelial cells, and if it is > 300 nm, the retention time in the cell will be longer and more effective^16–19^. On the other hand, acquitting the bloodstream after drug release is another crucial factor; the smaller compound would prepare the longer retention time in the bloodstream, which leads to more chances for finding the goal cells. In addition, the particle size should be under 200 nm for clearance from the bloodstream to be possible^20^. In this regard, this study has tried to optimize the particle size range of about 50–200 nm to improve the cellular internalizations and retention time^21,22^.

The influence of various chemical compounds on the tumor cells has been investigated and utilizing carriers has emerged in the last decade to deliver the therapeutic agents precisely. Among all nanocarriers, carbon-based materials such as C-dots^23^, rGO, and CNTs have shown the most promising properties due to their unique features like high surface per volume ratio, biocompatibility, non-toxicity, and low immunogenicity^24–28^. Carbon-based carriers like MWCNT (multiwall carbon nanotube) and rGO (reduced graphene oxide) have revealed second to none properties in the gene/drug delivery field because of their considerable biocompatibility, enhanced strength and conductivity, minor immunogenicity, high surface/volume ratio, great photothermal conversion at near-IR^29^, and uncomplicated functionalization^30^ which all these aspects can overcome different delivery barriers. This carrier feature’s climax would be that is low-cost and high-yield. These compounds are well-known as highly dispersible and stable in the aqueous media owing to the presence of abundant oxygenated functional groups such as carboxyl, hydroxyl, epoxy, and phenol on the edges as well as surface^31,32^. There are three methods for reducing the graphene oxide (GO) and deoxygenating it, i) Thermal, ii) Electrical, and iii) chemical methods, the third method (chemical) is widely accepted especially for large-scale synthesis. At this prosses, NaBH_4_, PEI, NH_2_NH_2_, Chitosan, and Resveratrol are usable^33^. Nowadays, rGO is well-known because of that although they have a favorable small size, they are capable to carry lots of various compounds for different missions. So, they opened up the window of opportunity to co-delivery systems^34^. Based on the literature, the carbon-based materials are considered a great controller of the systemic metabolism drug releaser and a good eliminable agent from the body which has minimal cytotoxicity^35–39^. Besides all advantages of carbon-based nanocarriers, they have some drawbacks such as partially drug-releasing before arriving at the tumor texture (uncontrolled release) and low capacity of drug loading (Table 1).

**Table 1:** Graphene-based nanosystem as a therapeutic carrier.

Content	Features (nm)	Tumor type	Test type		Drug	Specious		Properties	Results	Refs
			In vitro	In vivo		Human Animal				
GO/DOX/PEG-FA	100	Melanoma	B16F0	Male C57BL/6 J and Mice	DOX	*	*	Photothermal, photodynamic therapy and fl-imaging	Tumor treatment upon NIR light activation	^40^
GO/NPs/PvP	150 nm	Colorectaladeno carcinoma	Caco-2, HCT 116, NIH-3T3, HeLa, HEK-293 SCC-9		DOX	*		pH sensitivity	Safe for normal cell and toxic for cancerous cell	^40^
GO/AgNPs/Methotrexate	20 nm	Breast	MCF-7, Hep-G2		Methotrexate	*		Targeted therapy	Favorable particle size	^40^
rGO/CuInS_2_/ZnS/liposome	120 nm	Esophageal squamous carcinoma	Eca-109	Mice		*	*	Photothermal, photodynamic therapy	CuInS_2_/ZnS nanocrystals converted the laser beam to the heat and prepared ROS in the cell and decreased the toxicity	^41^
GO, GO/AgNPs, rGO, rGO/AgNPs		Lung	A549			*		Antitumor activity	rGO/AgNPs more cytotoxic than other, IC50: 30 µg/ml	^42^
Graphene Quantum-dots	65 nm		Dendritic cells					Immune response therapy	GQD can decrease the ROS creation	^31^
GQDs/Biotin	40 nm		A549		DOX	*		Targeted delivery	Lower toxicity	^41^

To circumvent the demerits of organic carriers, the porous ultra-low-density materials (metal–organic frameworks (MOFs)) are utilized which are an unequaled class of hybrid porous materials with distinctive features. These types of materials provide desirable facilities in the most important fields for humankind (gas storage^41^, luminescence^43^, nonlinear optics^44^, and gas separation^45^), especially at therapeutic agent’s uptake^46^, delivery^46^, and release^47^. All these properties originated from their specific characteristics^48^, such as tunable shape and pore size (up to six nanometers), functionalize-able pore and surface, and large surface/weight (up to 6240 m^2^/g), and adjustable composition. Among all prepared MOFs, some of them can satisfy the requirements of biomedical applications like size, stability, toxicological biocompatibility, required functional group on the surface, drug loading capacity, and drug-releasing just in the tumor’s acidic microenvironment. All the criteria mentioned above significantly influence delivery and encapsulation efficiency^49^. These organic/inorganic hybrid materials are formed by organic linkers and metals (metal chain, metal cluster, and single metal ion) self-assembly. The first and foremost thing for pharmaceutical and biomedical usage is choosing a toxicologically biocompatible building block. Based on being highly toxic, utilization of some metals (for example, Ni, Co, Cr, Sn, and Cd) as metal nodes are limited^50,51^. On the other hand, some metals exist in our bodies in large amounts, like the Fe in hemoglobin. The most suitable metals are Ti, Mn, Cu, Ca, Mg, Fe, and Zr, because of their presence in the biocatalytic processed of the living tissues and physiological systems^52^. These metals toxicity is screened by LD50 (oral lethal dose 50), and the results showed 25 g/kg (for Ti), 1.5 g/kg (for Mn), 25 µg/kg (for Cu), 1 g/kg (for Ca), 8.1 g/kg (for Mg), 30 g/kg (for Fe), 350 µg/kg (for Zn), and 4.1 g/kg for (Zr). There are two possibilities for choosing the linkers; one of them is utilizing exogenous linkers such as amines, imidazolates, pyridyl, and polycarboxylate. The LD50 toxicity data are reported: (2,6-napthalenedicarboxylic acid) 5 g/kg, (5-aminoisophthalic acid) 1.6 g/kg, (terephthalic acid) 5 g/kg, (isonicotinic acid) 5 g/kg, and (1-methylimidazole) 1.13 g/kg so these toxicities are acceptable in biomedical applications^53^. The MIL-100 (iron trimesic) and trimesic’s in vivo test have been screened and proved that these materials are utilizable in bio-applications^54^. Based on these collected data, the MIL125-(Ti) Ti_8_O_8_(OH)_4_(BDC)_6_ (BDC: benzene1,4-dicarboxylic acid) has been chosen because that is prepared by Ti as a metal nod and terephthalic acid as a linker and on the other side of the coin, this compound has excellent encapsulation capability, thermal stability, great adsorbability, and high porosity which make it a brilliant choice for drug delivery systems^55^.

Lots of medications (doxorubicin 32 mg/kg^56^, cyclophosphamide 251 mg/kg^57^, lomustine 70 mg/kg , carboplatin 343 mg/kg^58^, letrozole 2000 mg/kg^59^, paclitaxel 160 mg/kg , and trastuzumab 5110 mg/kg^60^) are usable for patients that are under chemotherapy and unfortunately many of them like DOX have severe side effects. These drugs can control some cancerous tissue criteria by connecting to the tumor’s overexpressed receptors^61^. Most of these drugs are injectable, and after injection, some links would be created between normal cells and the drugs, leading to cell death and lowering the efficiency of the drug. Among all antitumor medications, DOX is a highly toxic drug with wide-ranging applications that are utilized to treat Kaposi’s sarcoma^62^, breast cancer^63^, acute lymphocytic leukemia^64^, and bladder cancer^65^. Loading and controlled release of this drug is a controversial issue. So, employing a carrier that can deliver proficiently could be an intelligent approach (Table 2).

**Table 2:** Various outstanding metal–organic frameworks and their biomedical applications.

MOF Code	Metal NOD	Linker	Feature	Drug	Test type	Cell line	Ref
ZIF-8	Zn	Melm	One-pot process for encapsulating	α-TOS	in vitro and in vivo	HeLa, L929	^66^
Zn-GA	Zn	Glycolate	Without premature delivery	MTX	in vitro	PC12	^67^
HMS@ZIF	Zn	Imidazolate, Hollow mesoporous silica	-	DOX	in vitro	MCF-7	^68^
UIO-66	Zr	1,4-dicarboxybenzene	Designable layer-by-layer assembly	5-Fu	in vitro and in vivo	HeLa	^69^
MIL100-Fe	Fe	Trimesic acid	High stability at physiological pH	CPT	In vitro	HeLa, SH-SY5Y, Fibroblast 3T3	^70^
Zn- CPON-1	Zn	5-(40-carboxyphenoxy) nicotinic acid	Prediction of release kinetic behavior	5-FU	In vitro	HepG2, HASMC	^71^
Zr-MOF	Zr	Carboxylate	In situ polymerization strategy	Cisplatin	in vitro and in vivo	U87MG	^59^
UIO-68	Zr	4,4’-terphenyl-idicarboxylate	(MDA, 5 d, 55%), (MCF, 15%) appoptosis	CPT	In vitro	MDA-MB-231, MCF-10A	^42^
Fe-NDC	Fe	(H_3_BTB), (H_2_NDC)	Selective accum-ulation in CD44	Calcein and DOX	In vitro	HEK 293 T, MDA-MB-231, SCC7	^69^
HKUST-1	Cu	Benzene-1,3,5-tricarboxylate	11 d drug release	Nimesulide	-	-	^72^
NH_2_-Fe-BDC	Fe	Terephthalic acid	Co-encapsulated nanoformulation	DOX	In vitro	Panc-1	^57^
PCN-224	Zr	Porphyrin	Chemo and photodynamic therapy	DOX	In vitro	MDA-MB231	^41^

Transition metal complexes can act as a drug^73^ especially some of them which can mimic the Pt or Fe biological behavior in the physiological systems and are not so toxic. One of the most promising transition metals is Pd with singular features such as ability for mimicking the physiological mechanisms, being eco-friendly, and having low toxicity. Approximately all of the literature is considered the drugs as a ligand^74^ for preparing the complex while, practically most biocompatible materials with special substituents or functional groups such as (sulfhydryl, carbonyl, hydroxyl, amino, and methyl) can act as an overexpressed receptors blockers or apoptosis starter which both of these eventually lead to mutated cells death. Among all functional groups, carboxamide^75^ could be a good choice due to two major advantages: i) having both amino and carbonyl functional groups simultaneously which makes it one of the most vital functional groups in the nature and biomedicines and ii) connecting ability to the HER-2 and sigma 2 overexpressed receptors. These receptors are on the mutated cells' surface and they are responsible for cell proliferation, actually, the tumor's uncontrolled growth is due to their nature to overexpress their cellular structures. It has been proven that carboxamide-based ligands can hinder these receptors' proliferation signaling and as a result, it would be the end of cancerous cells' life^75^. There are several groups around the world that are reported the drug loading and release into/onto the MOFs but none of them has not investigated the effect of using a transition metal complex besides the drugs. The purpose of adding complex was to reinforce the amounts of hydrogen bonds and π-π interactions among the rGO, the MOF, and the drug that this claim will be investigated by the drug release testing.

As a result of considering the fore-mentioned data, an organic/inorganic nanocomposite designed by rGO, MIL-125(Ti), Pd(H_2_bpbenzo), and DOX and has been formed to conquer the limitations and nominate a new nanocarrier for delivering the anti-carcinogenic compounds precisely and accurately. Arranged nanocomposite has been employed in the co-delivery of DOX@PdL and the cellular and molecular investigation on HT-29 and HEK-293 cell lines (in vitro) and in vivo testing (liver toxicity index AST:ALT) has been performed as well.

## Materials and method

All utilized solvents and materials that were commercially available, has been purchased from Merck and Fluka and used as soon as opening. FT-IR (Fourier transform infrared spectroscopy), UV–Vis (Ultraviolet visible spectroscopy), EDS, Map and FESEM (Energy dispersive spectroscopy) (Field emission scanning electron microscopy), AFM (Atomic force microscopy), 2D Fluorescence Microscopy, ^1^H-NMR (Nuclear magnetic resonance), PXRD (Powder X_Ray_ diffraction), DLS and Zeta potential (Dynamic light scattering), AST and ALT liver index, and TEM (Transmission electron microscopy) analysis have been performed by flowing devices respectively: Unicam Maston 1000 FT-IR spectrophotometer (room temperature, 400–4000 cm^−1^, KBr pellets), Carrying 100 Bio Varian spectrophotometer (quartz cells,10 mm path length), MIRA3 TESCAN, ARA-AFM, FACSCalibur (BD, Germany), Bruker FT-NMR 500 MHZ spectrometer (^1^H-NMR of L and PdL, DMSO-d_6_ and CDCl_3_), PANalytical company X’Pert Pro MPD, HORIBA SZ-100, Selectra pro autoanalyzer spectrophotometer, and ZEISS em900.

## Synthesis section

### Synthesis of ligand H_2_bpbenzo (3,4-Bis(2-pyridinecarboxamido)benzophenone) (L)

Carboxamides were always synthesized in pyridine as a solvent (quite hazardous and highly toxic solvent)^72^ until last decade that ionic liquid synthesis method introduced^76^. This method is more environmental-friendly and greener. For preparing the H_2_bpbenzo (L), five mmol (1.06 g) 3,4-diaminobenzophenone, 10 mmol (1.23 g) 2-pyridinecarboxilic acid, were added to 10 mmol (3.10 g) triphenylphosphite and five mmol (1.61 g, TBAB) tetrabutylammonium bromide on 120 ˚C and harshly stirred for an hour and the product was viscose pale-yellow solid. At the next step, 10 mL methanol should be added to the cooled product and after 10 min stirring, the filtered precipitate would be the final product. It is better that wash this final product with cool methanol to eliminate the byproducts and not reacted compounds (Figure S1). L: **C**_**25**_**H**_**18**_**N**_**4**_**O**_**3**_ (422.45 g/mol): (Yield: 49%). Theoretical: C, 71.08; H, 4.29; N, 13.26. Practical: C, 70.09; H, 4.24; N, 13.06%. UV–Vis (DMF): λ_max_ (nm): 241, 332. FT-IR (KBr, cm^−1^): ν_max_: 3288 (s, N–H), 1495 (s, C–N), 1584 (s, C = C), 1697 (s, C = O_amidic_), 1655 (s, C = O_benzo_). ^1^H NMR (CDCl_3_, 400 MHz): δ (ppm) = 8.55, 8.64 (d, H_a,a´_) 10.22 and 10.66 (s, NH), 7.47–8.36 (14H, ArH).

### Synthesis of complex Pd^II^(bpbenzo) (PdL)

This complex has been synthesized by simple and one step method. In this regard, 0.25 mmol L: H_2_bpbenzo has been added to the solution of 0.25 mmol palladium acetate in 25 mL dichloromethane at room temperature and stirred for five hours. The mother liquor of filtered solution kept at cold and tranquil place. After about 5 days the yellow crystals has been obtained that were not appropriate for crystallography analysis (Figure S1). **C**_**25**_**H**_**16**_**N**_**4**_**O**_**3**_**Pd** (526.83): (Yield: 93%). Theoretical: C, 56.97; H, 3.08; N, 10.58. Practical: C, 55.55; H, 3.13 N, 10.30%. UV–Vis (DMF): λ_max_ (nm): 268, 387. FT-IR (KBr, cm^−1^): ν_max_: 1639 (s, C = O_amidic_), 1597 (s, C = O_benzo_), 1523 (s, C = C), 1511 (s, C-N). ^1^H NMR ((CD_3_)_2_SO, 400 MHz,): δ (ppm) = 8.668, 8.610(d, 2H, H_a,a´_), 8.603–7.023(14H, ArH).

### Synthesis of MIL-125(Ti)

This compound can be synthesized by different methods (reflux and solvothermal) and different precursors (Ti(BuO)_4_ and Ti(iPrO)_4_) which based on conducted studies, preferred method would be reflux and desirable precursor is Ti(BuO)_4_ due to higher crystallinity and phase purity (PXRD peaks matched far better with simulated pattern), higher surface area (BET surface area), more regular pore structure and distinct crystalline morphology^77^. All in all, the preparation of this compound started with dissolving 0.77 g terephthalic acid in 10 mL DMF in 100 mL round bottom flask which stirred for 30 min at 40 ˚C. At the next step, 2.8 mL methanol has been poured into the flask. After 30 min stirring, the solution has been refluxed for an hour at 100 ˚C. The last step of synthesis was adding precise amount of Ti(BuO)_4_ (0.84 mL) to the solution and the mixture has refluxed for 72 h at 110 ˚C. The prepared milky color precipitate separated utilizing centrifuge (14,000 rpm, 15 min) and collected product has been washed 3 time with methanol for decreasing the byproducts (Figure S1). (Yield: 67%), FT-IR (KBr, cm^−1^): ν_max_: 483 (s, Ti–O), 787 (s, C-H_aromatic_), and 1574 (s, C = C). PXRD (hkl): (101), (200), (212), (202), (302), (222), (312), (004), and (422).

### Fabrication of nanocomposite (rGO/MIL-125(Ti)@PdL@DOX)

**Figure 1:**
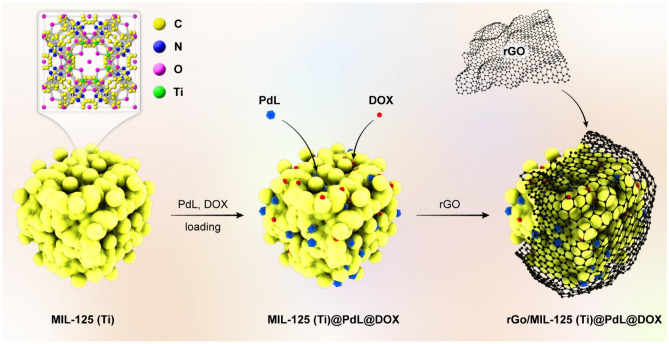
A schematic illustration of nanocomposite fabrication.

This study aimed to prepare a robust nanocarrier which has lower toxicity compared to toxicity of utilized compounds such as drug, MOF, and complex in the biological pH. Considering chemically deoxidation of GO, 200 mg of GO sonicated for an hour in 250 mL deionized water. To the suspended graphene oxide, 1.9 g of NaBH_4_ was added. Prepared mixture stirred for 10 h at STP condition and after that has been filtered and obtained black precipitate, has been severally washed and dried for five hours in 60 ˚C^78^. Then for restricting the DOX quenching, the next step has been done in a dark place and all beakers and sample containers covered by thick aluminium foil. Precise amount of DOX (5 mg) and PdL (650 mg) has inserted as a guest to the MIL’s cavities as a host (10 mL deionized water, 150 mg MIL-125(Ti)). Finally, for immobilizing the prepared compound on the nanocarrier, 120 mg of rGO has been added to the loaded MIL-125(Ti) container at room temperature (30 min stir), To removing unconnected compounds, the final nanocomposite centrifuged for 5 min at 10,000 rpm. All experiments were performed in accordance with the ARRIVE guidelines. A schematic illustration of the nanocomposite preparation in shown in Fig. 1.

### Cell culture

The process in which the cells can growth outside of their natural habitat under controllable conditions refers to cell culture. It is inevitable that different types of cells demand various circumstances. These various protocols utilize some similar medium, gases, and nutrients under special conditions (pressure, temperature, and pH). In practice, primary cells isolated directly from subject in growth media and then they can proliferate in the 96-well growth environment.

### MTT assay

The in vitro analysis was performed with ethical statement (IR.SBMU.RETECH.REC.1400.652) and approved by biomedical ethics committee in Shahid Beheshti University of Medical Sciences, Tehran, Iran. Two different cell lines have been applied, HT-29 (ATCC HTB-38) and HEK-293 (ATCC CRL-1721) (prepared from Pasture institute cells bank, Tehran, Iran). After growing the cells in DMEM (Dulbecco’s modified eagle medium) media supplements and 10% FBS (fetal bovine serum), the cells should be harvest by trypsin and utilizing the hemocytometer and trypan blue. At the next step, the cells should serially dilute and incubated by MTT solvent (3-(4,5-dimethylthiazol-2-yl)-2,5-diphenyl tetrazolium bromide) and then treated with samples for 24 and 48 h.

### Biological stability

In order to evaluating the nanocomposite stability in the different biological media, the same concentration of nanocomposites have been dispersed in the a: DI water, b: phosphate buffer saline (PBS), and c: DMEM + 10% FBS. The hydrodynamic radii have been monitored by DLS at 37 ˚C and various time point (0 – 48 h).

### pH adjusting and BSA adsorption

For adjusting the pH to the physiological pH, 2 mg/mL of prepared nanocomposite dispersed in DI water and then addition of 0.01 M NaOH adjusted the pH to the 11. At the next step, 2 mg/mL bovine serum albumin (BSA) added to the reaction flask and the mixture stirred for 5 min. The physiological pH adjusted by adding the 0.05 M nitric acid. The last step was addition of 100 µL of prepared suspension to the above-mentioned media. The DLS has been utilized to monitoring the nanocomposite’s stability^79^.

### In vivo* protocol*

The in vivo investigation was performed under the approved and routine protocols and according to the Guide for the Care and Use of Animals of Laboratory. We chose healthy mice to study the biocompatibility and safety of the synthesized nanocomposites. The male albino Wister rats were intravenously injected and were divided into different groups. After the injections, the mice were sacrificed and their liver was collected. The liver tissues were fixed in osmium tetroxide (1%), as well as the glutaraldehyde (2.5%) after collecting. It is quotable that, owing to safety evaluation, the liver toxicity index (AST:ALT) have been monitored. The collected samples dehydrated in epoxy resin and alcohols due to achieving to better comparison and images, we. For euthanasia of mice, the anesthetic overdose (CO_2_) has been utilized.

### AST and ALT (liver toxicity indexes)

A non-expensive, simple, and non-invasive test for predicting liver changes is the aspartate transaminase (AST) and alanine transaminase (ALT) screening. The AST and ALT enzyme investigation is the complement analysis of histopathological evaluation (H&E staining). In this regard, the injected mice blood enzyme was compared to the control mouse blood enzyme. Based on the literature, the AST:ALT ratio in the defected liver would be quite higher than the healthy liver AST:ALT ratio. The mice blood’s AST and ALT have been monitored by Selectra autoanalyzer spectrophotometer 24 h after intravenous injection (50 mg of samples).

### Histopathological evaluation

The synthesized samples have been excised and the 10% formalin was used for fixation (duration: one week, 2 times formalin replacement), the sulfuric acid has been utilized for decalcification of tissues, then, samples have been dehydrated in a ethanol serial dilution, embedded in paraffin, and Leica microtome sectioned the tissues to 5.0 µm thicknesses (Leica Microsystems, Germany). For all sections, Hematoxylin and Eosin (H&E) staining was performed and light microscope used for inspecting (Olympus, Tokyo).

### Antibacterial activity

In this investigation, conventional strains of *S. aureus*, a genus of gram-positive, and *P. aeruginosa*, a genus of gram-negative, were employed. Antibiotics from Muller–Hill Agar culture media were used to test the bacteria's susceptibility. Firstly, a solution containing a concentration of 10 g/mL of the substance in 0.01 mol/mL of HCl was produced. Following the incubation of bacteria, the plates were placed at 28 ℃ for 24 h. The disks are impregnated with a volume of 15 µL of rGO/MIL-125(Ti)@PdL@DOX, rGO/MIL-125(Ti)@PdL, and rGO/MIL-125(Ti)@DOX. Then, the discs were immersed in the medium and then placed in an incubator for 24 h at 28 ℃.

## Results and discussion

**Figure 2:**
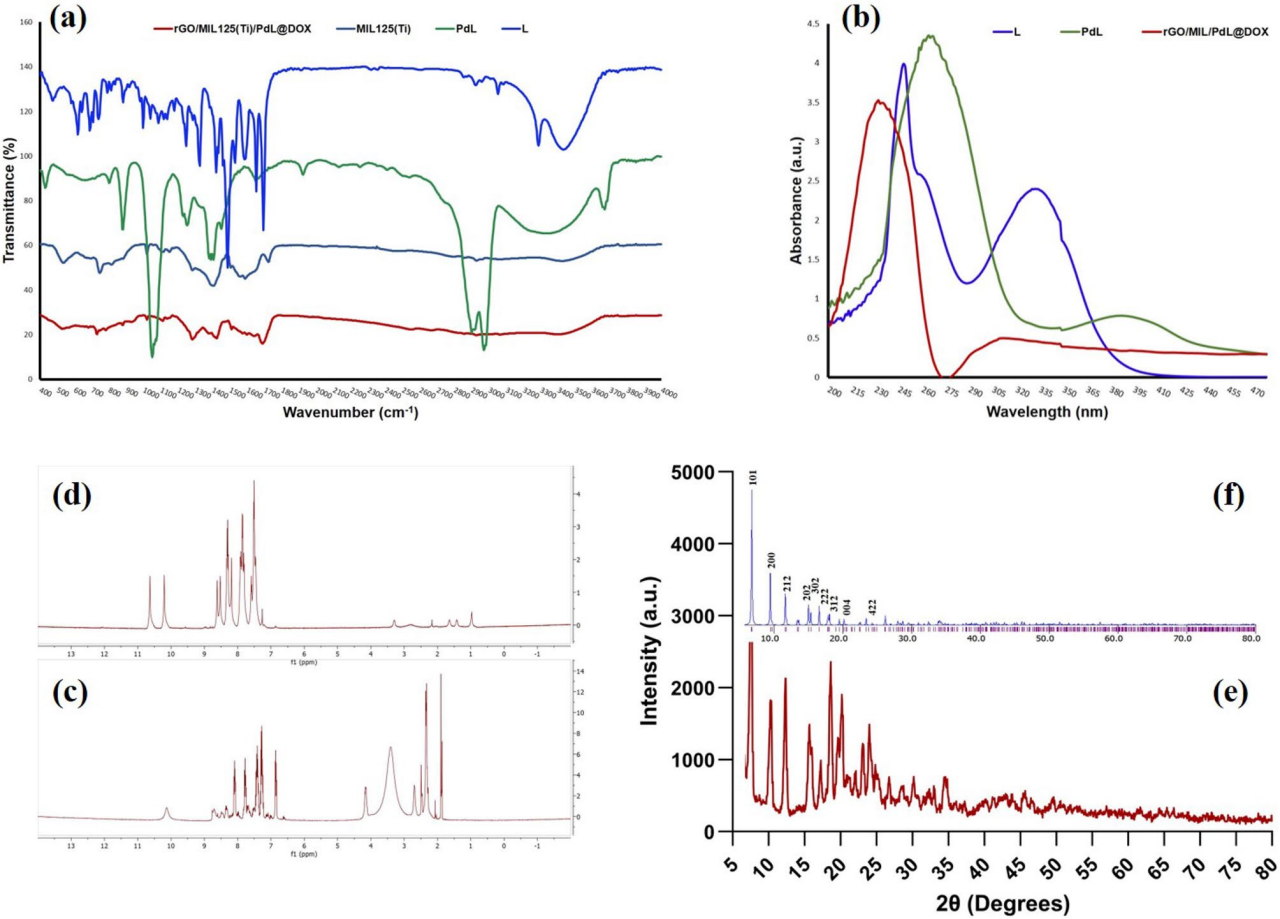
(**a**) The FT-IR spectra of; L: (3,4-Bis(2-pyridinecarboxamido)benzophenone), PdL: (Pd(3,4-Bis(2-pyridinecarboxamido)benzophenone)), MIL125(Ti), and nanocomposite: rGO/MIL-125(Ti)@PdL@DOX. (**b**) The UV–Vis spectra of; L, PdL, and nanocomposite. The ^1^H NMR spectra of: (**c**) PdL, (**d**) L. The PXRD spectra of **e**: prepared MIL-125(Ti) (**f**) simulated pattern.

The UV–Vis spectra of prepared materials in dichloromethane are showed in Fig. 2b. The L: (H_2_bpbenzo) bands are revealed at 240, 263, and 330. All these three bands assign to the intra-ligand transitions such as aromatic rings π → π* and the nitrogen electron pair’s n → π*. The two bands of PdL centered at 268 (broad band) and 387 nm that are related to n → π*, π → π* (intra-ligand transitions), and charge transfer. The nanocomposite (rGO/MIL-125(Ti)@DOX@PdL) just has one peak at about 228 nm that is related to DOX transitions. The FT-IR spectra has been showed at Fig. 2a, the L’s: (3,4-Bis(2-pyridinecarboxamido)benzophenone) spectra have a lot of peaks due to diversity of it functional groups and the most important peaks are as follows: the C = O_benzo_, C = O_amidic_, and C = C stretching mode are represented at about 1655, 1697, and 1584 cm^−1^ respectively. The peak which appeared at 3288 is related to N–H. The amidic C = O bond presence is proved by a sharp band at about 1697 cm^−1^ and the C-N bonds peak revealed at 1520 cm^−1^. The N–H bonds peak is disappeared at the spectra of PdL’s: (Pd(3,4-Bis(2-pyridinecarboxamido)benzophenone)) as a results of coordination to the metal (Pd) and deprotonation, which could be the first evidence of correct synthesis of complex. The peak of amidic C = O appeared at 1697 cm^−1^ and it is red shifted to the 1597 cm^−1^ in the spectra of complex. Another confirmation for deprotonation and correct connection of nitrogen to the Pd would be the blue shift of ν_C-N_ from 1495 to 1511 cm^−1^ and it is due to C-N bond strengthening and resonance enhancement. These compounds FT-IR spectra are in a good concurrence with other literature^80,81^. The MIL-125(Ti) peaks are appeared at 483, 787, and 1574 cm^−1^ that are related to the connection of titanium to the oxygen, aromatic C-H bond, and aromatic C = C respectively. Because of presence of H_2_O in the utilized solvent, the broad peak has been viewed at around 3400 cm^−1^. The nanocomposite has revealed MIL-125(Ti)-like FT-IR spectra because of exitance of a lot of MIL-125(Ti) on the nanocarrier^82^.

**Figure 3:**
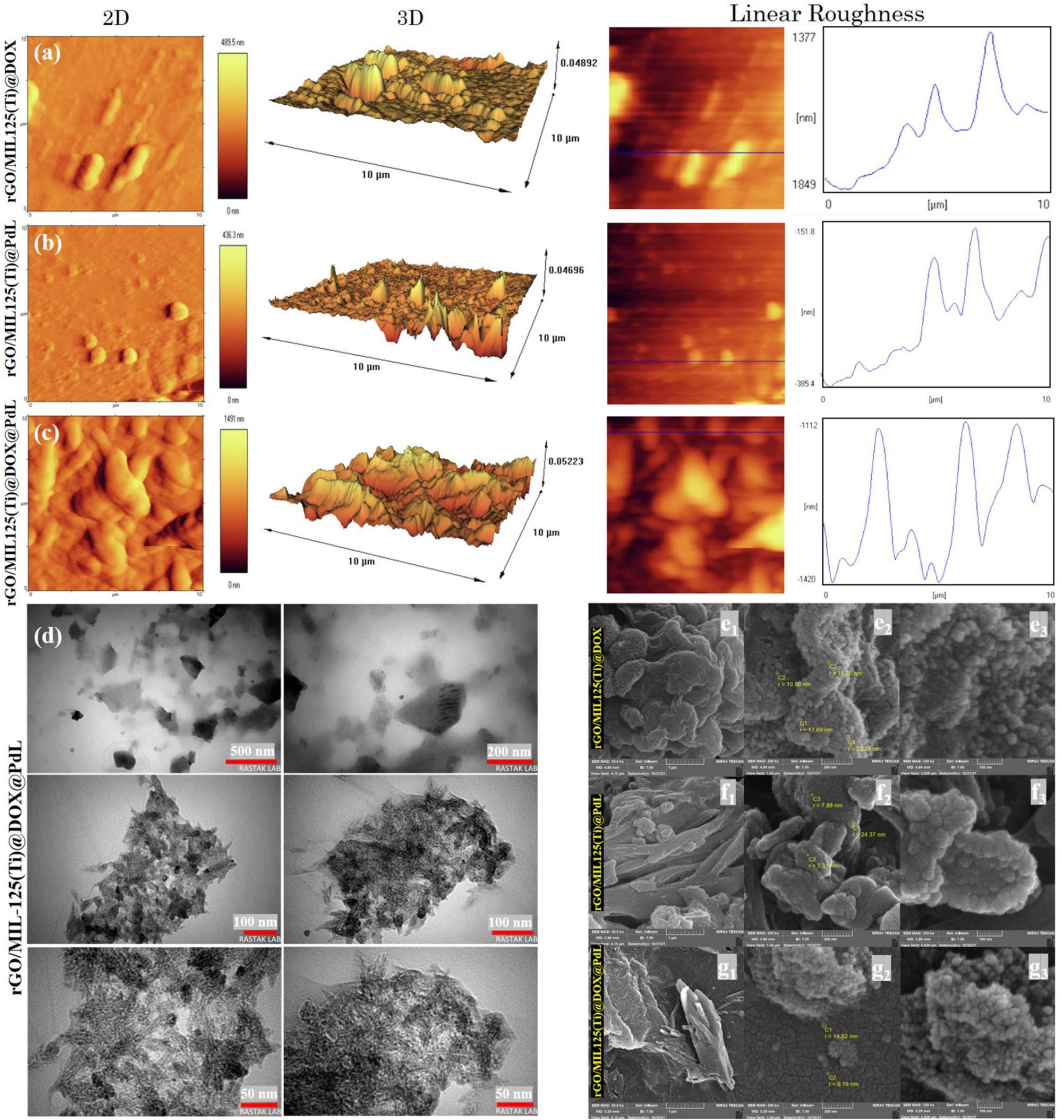
The 2D, 3D, and linear roughness of nanocomposites’ poriferous surface (**a)** rGO/MIL-125(Ti)@PdL, **(b)** rGO/MIL-125(Ti)@DOX, and (**c**) (rGO/MIL-125(Ti)@PdL@DOX). (**d)** TEM results of prepared nanocomposite rGO/MIL-125(Ti)@DOX@PdL. FESEM results of prepared nanocomposites. (**e)** rGO/MIL-125(Ti)@DOX, (**f**) rGO/MIL-125(Ti)@PdL, (**g)** rGO/MIL-125(Ti)@DOX@PdL.

The L and PdL’s ^1^H-NMR spectra have been measured in deuterated chloroform and reported in Figs. 2d and c. The most important peak in this type of ligands and complexes is the peaks of amidic protons (N–H) which reveal at around 10–11 ppm. The aforementioned peaks should disappear after the accurate connection between nitrogen and Pd. As it can be seen, these peaks have vanished in the PdL ^1^H-NMR spectra which is reliable evidence of complex synthesis. Based on the conducted studies, the peaks of quinolone’s hydrogen must be appeared at about 7.00–9.00 ppm, and they are revealed at 7.28–8.73 ppm for L and they are blue-shifted in the PdL spectra owing to changing the symmetry, angles, and bonds length. The most important Hydrogens of ligand are labeled in Figure S15. All these peaks are in good agreement with published research^83^. The PXRD pattern of prepared MIL-125(Ti) was in acceptable agreement with the simulated pattern and previously conducted survey. Presence of a quite sharp peak at about 2Ɵ: 7˚ revealed that the synthesized material was crystalline (Fig. 2e and f). The surface morphology of prepared nanomaterials has been evaluated by AFM (Atomic force microscopy analysis) and demonstrate (Fig. 3a–c) that utilizing PdL beside DOX leads to the higher poriferous surface which increase the drug loading capacity. The exact amount of roughness is not apparent, but, roughness relative amount would be promising for comparing the prepared nanocomposites with other reports. Hitherto, it is evident that the roughness of pristine nanocarrier is quite lower than nanocomposites and based on collected data in this study, using DOX and PdL beside each other leads to higher roughness in comparison with using them separately (Fig. 3a–c). These finding are agreed by electron microscopy (Fig. 3d–g) and literature.

**Figure 4:**
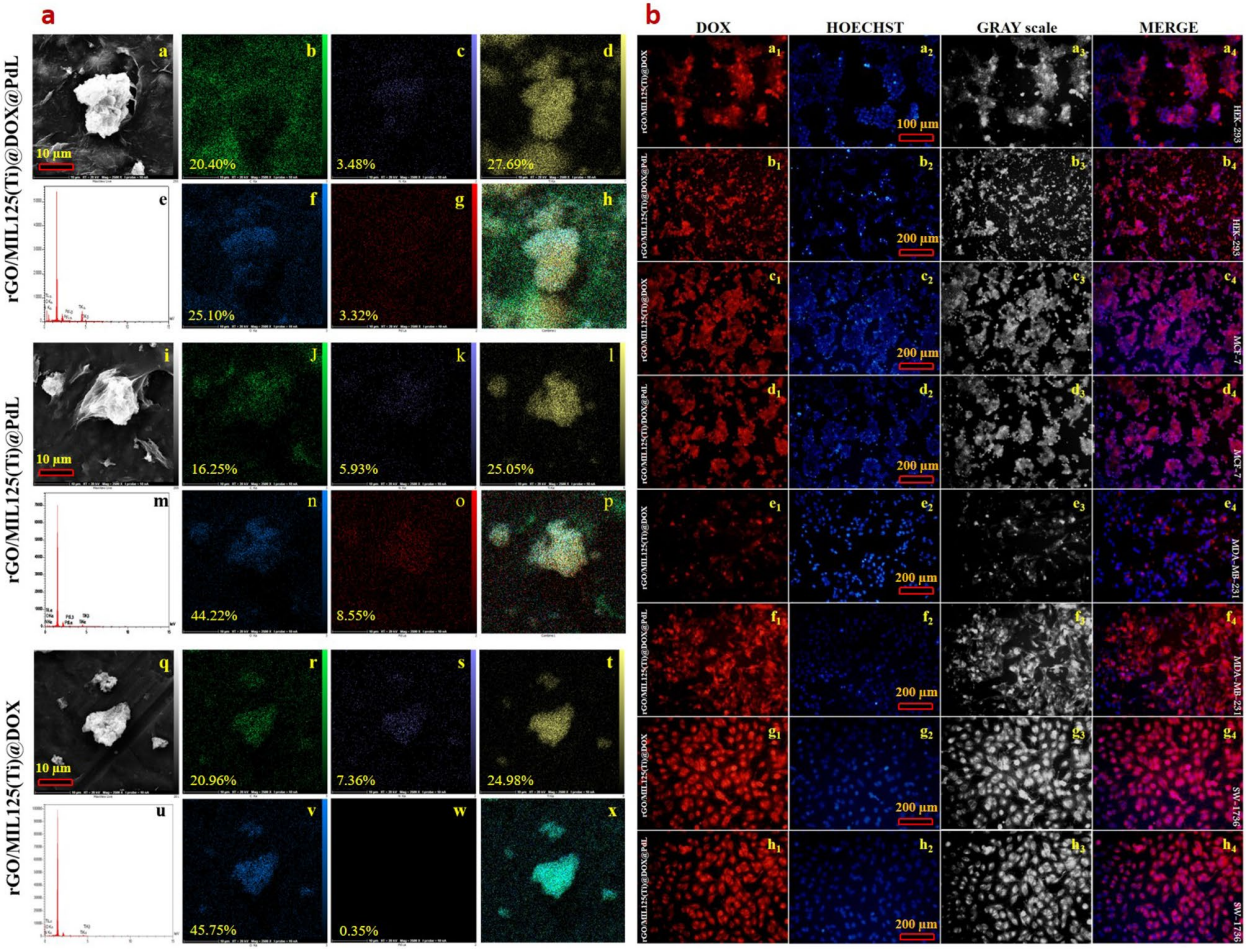
(**a)** The EDS and map of rGO/MIL-125(Ti)@DOX@PdL (*a-h*), rGO/MIL-125(Ti)@PdL (*i-p*), and rGO/MIL-125(Ti)@DOX (*q-x*) nanocomposites. (Max view: *a, i,* and *q*. Carbon: *b, j,* and *r.* Nitrogen**:** *c, k,* and *s.* Titanium: *d, l,* and *t.* Oxygen: *f, n,* and *v.* Palladium: *g, o,* and *w.* Combination: *h, p,* and *x*. EDS: *e, m,* and *u*). (**b)** Intracellular uptake images of DOX loaded nanocomposites by 2D fluorescence microscopy on HEK-293 (*a* and *b*), MCF-7 (*c* and *d*), MDA-MB-231 (*e* and *f*), and SW-1736 (*g* and *h*) cell lines (treatment time was 4 h).

It would be noticeable that, the more roughness on the nanocomposite surface, the higher ability to connect which is favorable in the drug release Sect.^84–86^. The fabricated nanocomposites' 3D and 2D images are shown in Fig. 3a–c, which can confirm the abovementioned idea. To obtain the structural information and microscopic morphology, TEM analysis of the prepared nanocomposite (rGO/MIL-125(Ti)@DOX@ PdL) has been carried out. The images of this analysis clearly illustrated that the maximum size of prepared nanocomposite is 190 nm that makes this nanocomposite markedly applicable in drug delivery system, clarifying this, based on reported biological investigation the nanocarrier's size should be in the range of 10–200 nm for accurate finding targeted cell, easily entering, enough remaining, and properly cleansing (Fig. 3d)^87,88^. The obtained sample morphology has been observed and presented for comparison in Fig. 3e–g. As it can be seen, the reported FESEM images reveal a larger semi-spherical shape for rGO/MIL-125(Ti)@PdL and semi bipyramidal shape for rGO/MIL-125(Ti)@DOX@PdL and rGO/MIL-125(Ti)@DOX which demonstrate that combination of PdL with MIL-125(Ti) might lead to the new bond between Ti nods and PdL. Using DOX lonely can lead to a little bit of aggregation for MIL-125(Ti), and utilizing it beside PdL could not noticeably change the MIL-125(Ti) morphology owing to linking the L’s quinolines aromatic rings to the DOX aromatic ring by π-π interaction. All patterns have been shown to have a carbon-based substrate and single-layer nanomaterials structure. It is proved that this type of aggregation which is illustrated in Fig. 3e1, f1, and g1 does not have any effective problem in biomedical usage (gene/drug delivery)^69,89^. The SEM–EDS analysis was utilized for the characterization of the prepared samples' chemical composition. Figure 4a, illustrate that the synthesized nanocomposite surface contains carbon, palladium, titanium, nitrogen, and oxygen. The EDS mapping of these elements has displayed that these elements are well distributed on the surface of the nanocomposite. As it can be seen, the oxygen percentage is far more than the nitrogen percentage which these differences would make the surface potential negative and the zeta potential analysis result has been proved it (zeta potential: -10 mV) (Figure S16). The investigation of these images can shed a clear light on understanding surface morphology. First of all, the shape of the MIL-125(Ti) is semi-spherical in the max view of the rGO/MIL-125(Ti)@PdL, and the shape of this compound in the max view of rGO/MIL-125(Ti)@DOX@PdL and rGO/MIL-125(Ti)@DOX is semi-bipyramidal. Secondly, most of the palladium and nitrogen distributions are centralized on/in the MIL-125(Ti). It is noticeable that the oxygen saturation on the rGO surface is quite lower than MIL-125(Ti) surface^90^.

The stability investigation before any biological analysis is necessary for any new nanomaterials’^91–93^. In this regard, the biological stability of synthesized nanoparticles has been evaluated in different media for 2880 min (Fig. S14A and B). The nanocomposite’s particle size in the DMEM + 10% FBS (Figure S14B) has been doubled (compared to the median DLS results of nanocomposite in the DI water) after pouring the DMEM + 10% FBS to the pH-adjusted mixture and then increased to about 600 nm near 48 h. Based on reported results, its increment trend would be plateaued after 48 h^94^. The nanocomposite’s particle size in the PBS was increased by about 30% immediately after medium addition and then plateaued before the 24 h (~ 250 nm). Based on the literature, these collected data revealed acceptable biological stability when some of the reported nanomaterials were aggregated and not stable for longer than 24 h^95,96^. To achieve the sharp and clear images of the cells, fluorescence microscopy has been utilized instead of conventional microscopes and it is due to excluding most of the lights from samples that are not eradiated from microscopes. It should be noted that the encapsulation efficiency of DOX to the prepared nanocomposite was recorded at 48.9%. The conventional microscopes are not utilizable for viewing the thick tissue specimen for in vitro and in vivo investigation owing to requiring a thin cut of frozen or fixed tissue^97^. Regarding, the cellular uptake has been carried out by 2D fluorescence microscopy before any in vitro investigation of the cultured cells. The DOX can interact chemically and physically into and onto the nanocarrier's porosity (van der Waals interactions, hydrogen bonds, and π-π interactions). Additionally, this study’s engineered nanosystem can establish more π-π interactions between PdL and DOX. This reinforced connection leads to lower cytotoxicity in the biological pH which is vivid in the 2D fluorescence microscopy and MTT assay results (HEK-293 cell line). The images of the 2D fluorescence microscopy (Fig. 4b) demonstrate that the nanocomposites are connected successfully to the cell membrane instead of the cell nucleus. The cells population prove the fact that the prepared nanomaterial's cytotoxicity is not high and this makes them a competent candidate for drug and gene delivery systems. The MDA-MB-231 cell line is quite susceptible to the DOX and as a result of its easier leaching from rGO/MIL-125(Ti)@DOX in comparison with rGO/MIL-125(Ti)@DOX@PdL, the cell population of that (Fig. 4b–e1) is conspicuously dropped.

**Figure 5:**
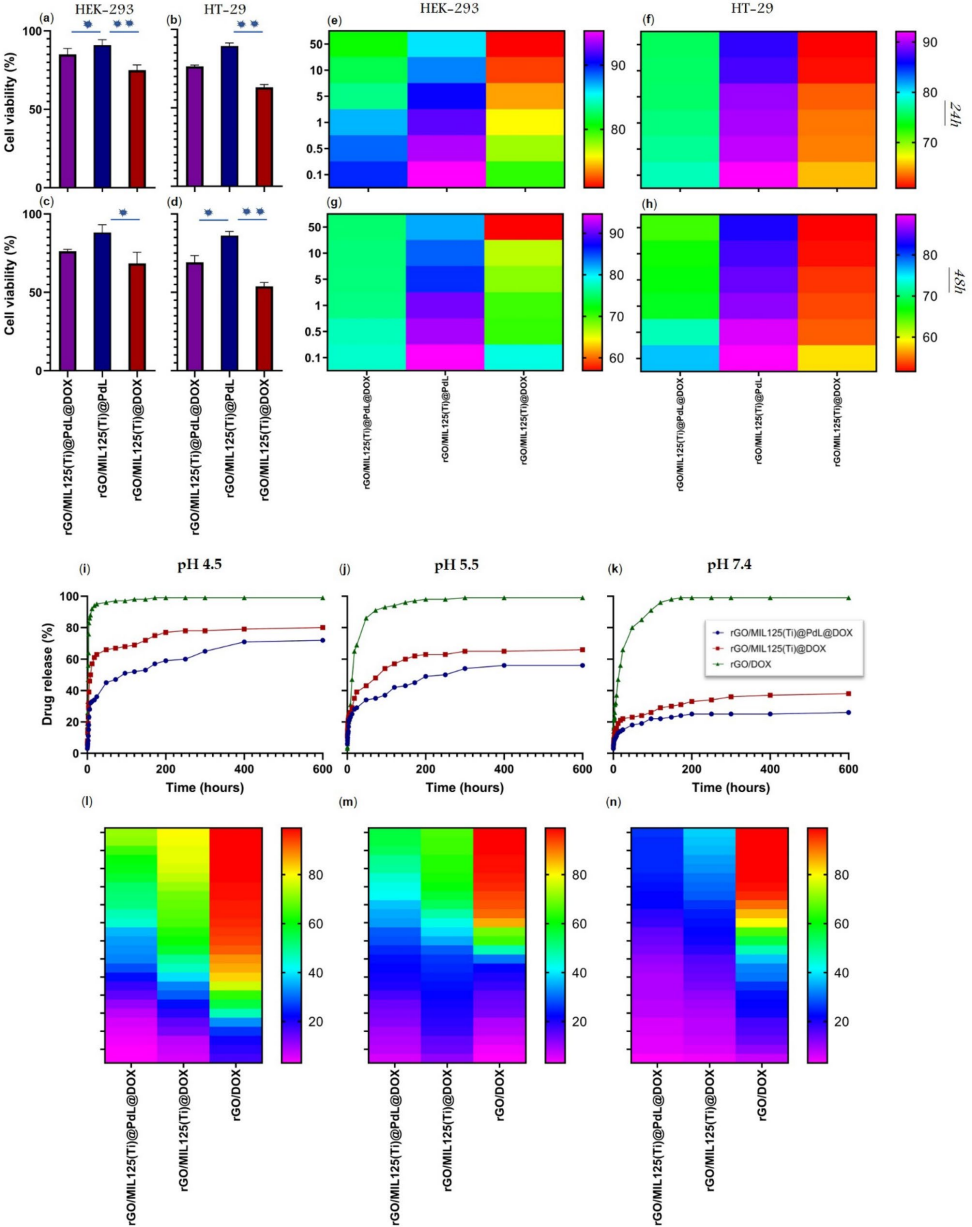
The MTT assay median results after 24 and 48 h of treatment on the HEK-293 (**a** and **c**) and HT-29 cell lines (**b** and **d**). The MTT assay’s heat map results in different concentrations (0.1–50 mg/mL) of different nanocarriers and nanomaterials on the HEK-293 (**e** and **g**) and HT-29 (**f** and **h**) after 24 and 48 h of treatment. The drug release profile (time-dependent characteristic) at pH (4.5), (5.5), and (7.4) showed in **i, j,** and **k** respectively. The 2D heat-map at pH (4.5, 5.5, and 7.2) drug release profile has been revealed at (**l, m,** and **n**). (The bluish and the reddish color can indicate the lowest and the highest drug release percentage). *p value < 0.05 and **p value < 0.01.

The following-up action after successful cellular uptake would be screening the cytotoxicity of the specimen on the cell lines. All fabricated nanosystem toxicity has been carried out on the HEK-293 cell line and the HT-29 cell line. Based on the MTT assay results, although, both cultured cells demonstrated a similar and convergent manner after 24 and 48 h treatment, HT-29 (Fig. 5b, d, f, and h) cell line revealed more sensitivity (about 50%) against DOX compared to HEK-293 (Fig. 5a, c, e, and g). The median cytotoxicity of rGO/MIL-125(Ti)@DOX on the HEK-293 were 25% and 31% for 24 and 48 h treatment (respectively) and for HT-29 were 36% and 45% and it is scientific and proven fact that more than 30% toxicity have a catastrophic consequence on eukaryotes. The cell viability of rGO/MIL-125(Ti)@PdL was excellent on both cell lines and the MTT results illustrated only about 10% cytotoxicity for this nanocomposite, it is quotable that based on the other survey, the complexes which prepared by palladium and carboxamide have a high cytotoxicity (about 30%) and it is quite interesting that inserting this high toxic compound to the MIL-cage has been drastically increased the cell viability. As in the last sections it (utilizing a complex of palladium beside DOX) showed that can be profitable, and it is time to turn our attention toward its MTT assay results. As it can be seen, the cytotoxicity of rGO/MIL-125(Ti)@DOX@PdL on HEK-293 cell lines were 15% and 24% for 24 and 48 h treatment (respectively) and for HT-29 cell lines were 23% and 30% that these data are about 50% batter than utilizing DOX lonely, it is absolutely appealing that this study’s aim fulfilled and the more-biocompatible compound has been designed. The correlation between treatment time and concentration and their effect on the cell viability has been illustrated on the heat map graph (Fig. 5e–h), and by increasing the concentration of nanocomposites and spending the time, the cell viability has been decreased. The interaction among the prepared nanostructures and cell membrane explanation needs more biological experiments. The three parameters’ statistic results of these calculated data are demonstrated in the Table S2-S5. The screening of IC50 results showed reverse ratio between the treatment duration and the concentration of nanocomposite for utilizing just PdL in the nanocomposite structure, it means by doubling the time of treatment, the IC50 halved. On the other hand, after doubling the treatment time for a sample with just DOX, the IC50 become a tenth which is due to the high toxicity of DOX. At the end, the IC50 value of rGO/MIL-125(Ti)@DOX@PdL relatively quartered by doubling the treatment time.

As a proven fact, the tumorous cellular endo-lysosomes have lower pH in comparison with normal cells, to mimic this acidic condition and investigating this condition effect on the DOX loaded nanosystem, three different nanomaterials (rGO/DOX, rGO/MIL-125(Ti)@DOX, rGO/MIL-125(Ti)@DOX@PdL) have been used in a buffered solution at pH: 4.5, 5.5, and biological pH 7.4 (Fig. 5i–k). This analysis results revealed that in the absence of any type of connection reinforcer (MIL-125(Ti) and PdL), the DOX release would be spontaneous and even in the biological pH (7.4), about 100% of DOX would be drastically released before 120 h and it would be severe by decreasing the pH to the 4.5 which almost 100% of DOX discharged from nanocarrier before 20 h because of easily protonation and increasing the solubility of DOX. Presence of MIL-cage and PdL lead to superior adsorption and absorption of DOX due to porous surface, cavities, and π-π interactions which prevent the DOX’s readily off-load. As it can be seen in the Fig. 5l–n, although both of the rGO/MIL-125(Ti)@DOX and rGO/MIL-125(Ti)@DOX@PdL had an acceptable drug release in the physiological pH 7.4, as it was expected, the release-percentage of rGO/MIL-125(Ti)@DOX@PdL was about 40% lower than rGO/MIL-125(Ti)@DOX that is considerable. The desirable nanosystem should release the therapeutic agent step by step and gradually, this manner is more vivid at the trend of rGO/MIL-125(Ti)@DOX@PdL and it would be on account of nanocarriers harder fragmentation because of more π-π stacking interaction among rGO, MIL, PdL, and DOX. All the release process can ascribe to the decreasing the nanocarrier surface negative charge due to effortlessly protonation of the nanocarriers surface in the acidic pH which leads to decreasing the electrostatic interaction among the nanocarrier and the other protonated compounds that are immobilized on the nanocarrier. The collected results screening revealed that the proposed nanocomposite (rGO/MIL-125(Ti)@DOX@PdL) has a dependent response to tumoral pH and can release the therapeutic slowly but surely^98,99^.

**Figure 6:**
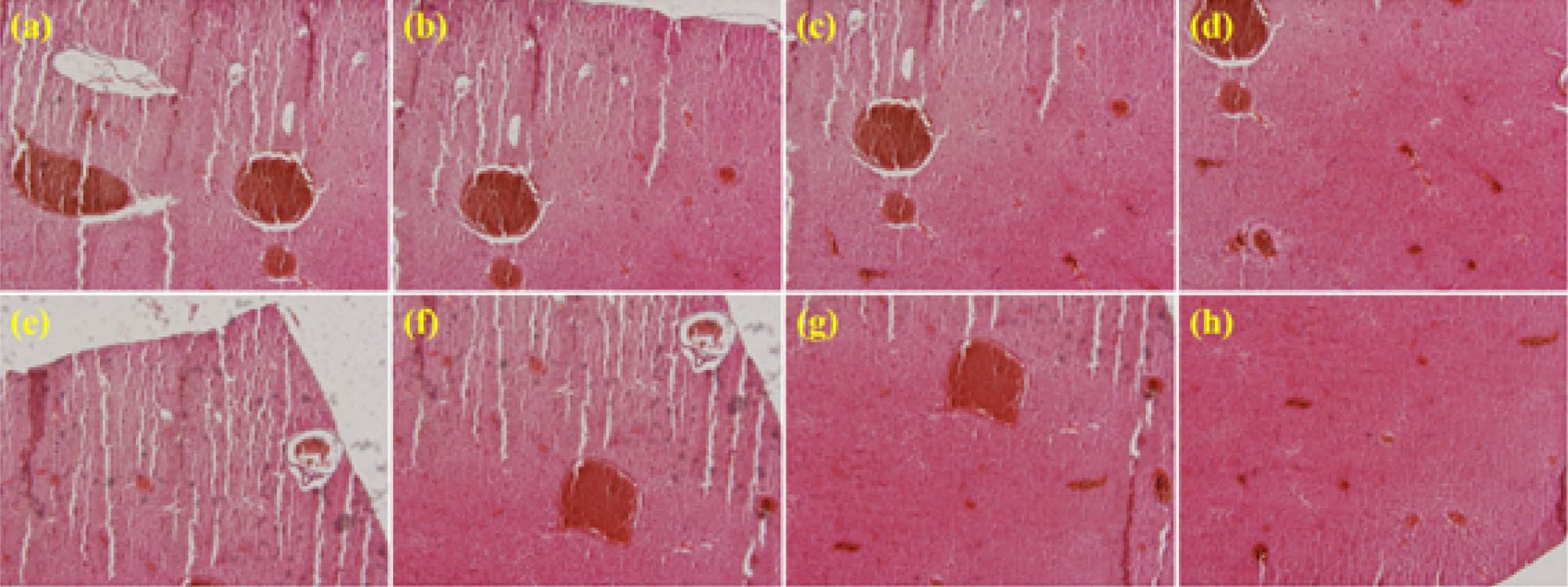
High magnification of H&E counterstain of (**a-d)** rGO/MIL-125(Ti)@DOX@PdL, and **(e–h)** rGO/MIL-125(Ti)@DOX on rat liver.

In order to investigate the in vivo biocompatibility and effect of the prepared nanoparticles on the liver organ, H&E analysis on the isolated livers proceeded and the morphology of the liver after injections was evaluated. Based on the results (Fig. 6), some sites of the liver were degraded because of the presence of toxic compartments in the injected sample; however, the whole morphology of the liver remained intact, which is a promising result. The AST and ALT liver toxicity index have been monitored as a histopathological complimentary analysis. Based on the collected data and compared to the literature, the AST:ALT ratio for healthy mice is under 1.5 and our AST0:ALT0 ratio for the control mouse was 1.32. As we had the control sample, every ASTn:ALTn ratio should be calculated as follow: ASTn:ALTn/AST0:ALT0 (n is the sample number). In this regard, the result of intravenous injection of 50 mg of rGO/MIL-125(Ti)@DOX after 24 h was 2.06/1.32 = 1.56 and this ratio for rGO/MIL-125(Ti)@DOX@PdL was 1.93/1.32 = 1.46 which is another evidence for biosafety of prepared nanocomposite^100,101^.

The prepared nanocomposites’ (rGO/MIL-125(Ti)@DOX@PdL) antibacterial activity has been screened and compared with rGO/MIL-125(Ti)@PdL and rGO/MIL-125(Ti)@DOX. These nanocomposites' antibacterial activity has been tested against *P. aeruginosa* as a gram-negative and *S. aureus* as a gram-positive bacterium and the median results are reported. The pictures in Figure S2 can successfully vivid that all these nanocomposites had acceptable inhibitory against both gram-positive and -negative bacterium. Based on other conducted surveys, it was expectable that between rGO/MIL-125(Ti)@PdL (Figure S2-b^+^ and S2-b^−^) and rGO/MIL-125(Ti)@DOX (Figure S2-c^+^ and S2-c^−^), the PdL containing nanocomposite reveal more inhibitory activity owing to preparing more ROS (reactive oxygen species) which has been fulfilled. The synergic effect of utilizing DOX and PdL beside each other made considerable differences. Using PdL beside DOX has increased (about 50%) the antibacterial activity on gram-negative and about 200% on gram-positive bacterium which was quite promising (Figure S2-a^+^ and S2-a^−^). It is noticeable that all tests have been repeated three times and the median data are reported. These considerable results showed that not only prepared nanocomposites to have antibacterial activity, but also utilize palladium complexes beside the drug can boost inhibitory activity.

## Conclusion

Based on structural engineering, the rGO, MIL-125(Ti), PdL, and DOX have been chosen to achieve a more efficient, environmentally friendly, biocompatible, biodegradable, and cost-effective nanosystem (rGO/MIL-125(Ti)@DOX@PdL). Subsequently, prepared nanocomposite has been characterized by TEM, FESEM, ^13^C,^1^HNMR, UV–Vis, FT-IR, and AFM. The cytotoxicity of rGO/MIL-125(Ti)@PdL@DOX has been screened by different cell lines (HEK-293 and HT-29) and imaged by 2D fluorescence microscopy and the drug release profile (time-dependent) of fabricated nanomaterial in various pH (4.5, 5.5, and 7.4) carried out. Antibacterial activity screening revealed the most promising results and showed that utilizing PdL next to the DOX can extend the inhibitory activity by about 200%. The in vivo testing was conducted on rat liver and H&E results were imaged by 2D fluorescence microscopy. The in vivo results showed more than 60% biocompatibility with the liver of the mammalian, which is a promising result. The goal of this exploration was synthesizing the green and efficient nanocomposite which is utilizable for codelivery of PdL and DOX which has been fulfilled.

## Supplementary Information

Supplementary Information.

## Data Availability

All data generated or analyzed during this study are included in this published article.
