# Supplementary material for: MIL-125-based nanocarrier decorated with Palladium complex for targeted drug delivery
Source: Sci Rep. 2022 Jul 15;12:12105. doi: 10.1038/s41598-022-16058-w (PMC9287414; doi:10.1038/s41598-022-16058-w)
Supplement: Supplementary file 1 — Supplementary Information. [file 41598_2022_16058_MOESM1_ESM.docx]

**Supporting information**

**MIL-125-based nanocarrier decorated with Palladium complex for targeted drug delivery**

*Mojtaba Bagherzadeh^1^*, Moein Safarkhani^1^, Mahsa Kiani^1^, Fatemeh Radmanesh^2,3^, Hossein Daneshgar^1^, Amir Mohammad Ghadiri^1^,* *Fahimeh Taghavimandi^1^, Yousef Fatahi^4,5,6^, Nahid Safari-Alighiarloo^7^,* *Sepideh Ahmadi^8^, Navid Rabiee^9,10^*

1. *Department of Chemistry, Sharif University of Technology, Tehran, Iran*
2. *Uro-Oncology Research Center, Tehran University of Medical Sciences, Tehran, Iran*
3. *Department of Stem Cells and Developmental Biology, Cell Science Research Center, Royan Institute for Stem Cell Biology and Technology, ACECR, Tehran, Iran*
4. *Nanotechnology Research Centre, Faculty of Pharmacy, Tehran University of Medical Sciences, Tehran, Iran*
5. *Department of Pharmaceutical Nanotechnology, Faculty of Pharmacy, Tehran University of Medical Sciences, Tehran, Iran*
6. *Universal Scientific Education and Research Network (USERN), Tehran, Iran*
7. *Endocrine Research Center, Institute of Endocrinology and Metabolism, Iran University of Medical Sciences, Tehran, Iran*
8. *Department of Medical Biotechnology, School of Advanced Technologies in Medicine, Shahid Beheshti University of Medical Sciences, Tehran, Iran*
9. *School of Engineering, Macquarie University, Sydney, New South Wales, 2109, Australia*
10. *Department of Materials Science and Engineering, Pohang University of Science and Technology (POSTECH), 77 Cheongam-ro, Nam-gu, Pohang, Gyeongbuk, 37673, South Korea*

*Corresponding author: Prof. Mojtaba Bagherzadeh ([bagherzadeh@sharif.edu](mailto:bagherzadeh@sharif.edu))


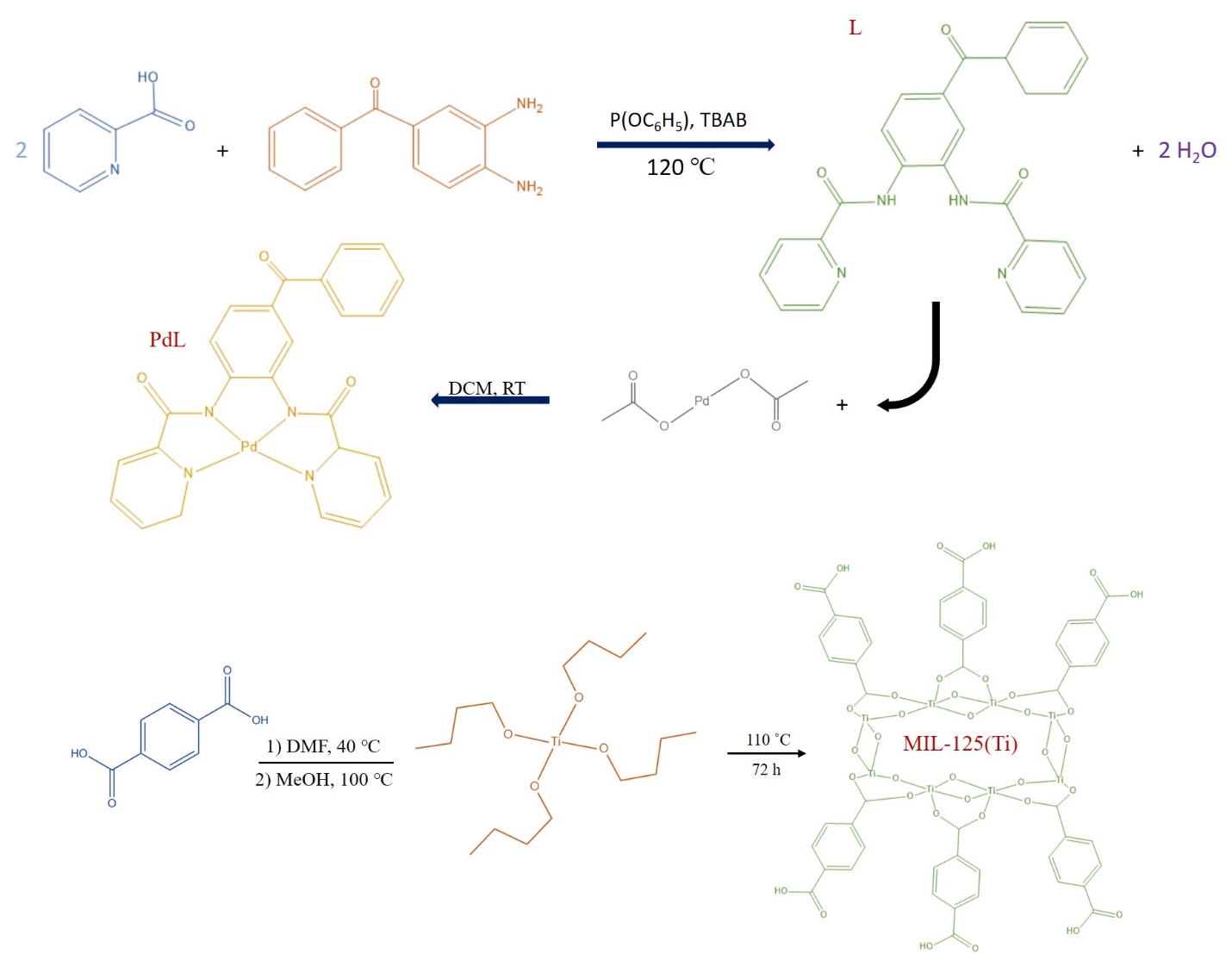


**Figure S1.** Schematic illustration of synthesis of L, PdL, and MIL-125(Ti)


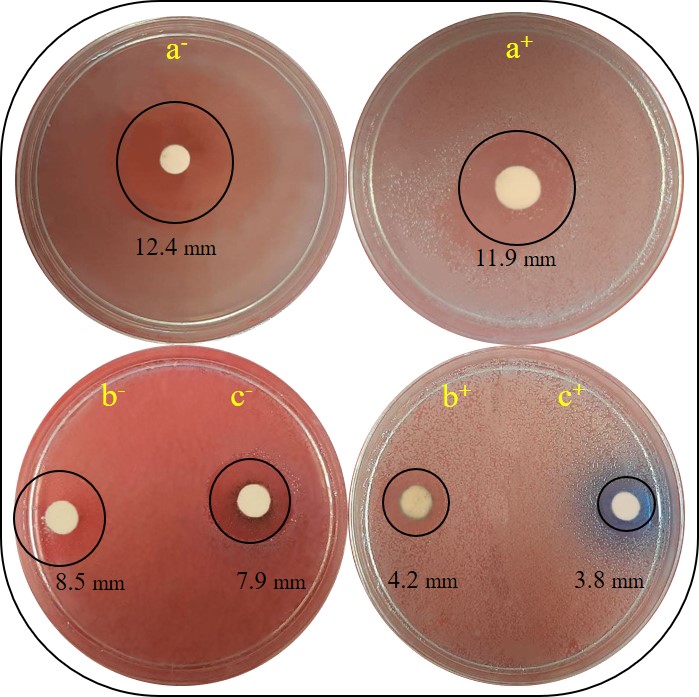


**Figure S2.** The antibacterial disc-diffusion results of **a^-^:** rGO/MIL-125(Ti)@PdL@Dox**, b^-^:** rGO/MIL-125(Ti)@PdL, and **c^-^:** rGO/MIL-125(Ti)@Dox on gram-negative bacteria (*P. aeruginosa*) and **a^+^:** rGO/MIL-125(Ti)@PdL@Dox**, b^+^:** rGO/MIL-125(Ti)@PdL, and **c^+^:** rGO/MIL-125(Ti)@Dox on gram-positive bacteria (*S. aureus*)*.*

**Table S1.** The IC50 results of prepared nanocomposites.

| IC50 value (mM) | **rGO/MIL-125(Ti)@PdL@Dox** | **rGO/MIL-125(Ti)@PdL** | **rGO/MIL-125(Ti)@Dox** |
| --- | --- | --- | --- |
| HEK-293 24h | 2.22 | 4.11 | 1.28 |
| HEK-293 48h | 0.51 | 2.05 | 0.12 |
| HT-29 24h | 0.44 | 3.42 | 3.33 |
| HT-29 48h | 0.36 | 1.29 | 0.16 |

**Table S2.** The IC50 results of HEK-293 24h

| (Inhibitor) vs. response (three parameters) | rGO/MIL125(Ti)@PdL@Dox | rGO/MIL125(Ti)@PdL | rGO/MIL125(Ti)@Dox |
| --- | --- | --- | --- |
| **Best-fit values** |  |  |  |
| Bottom | 80.14 | 85.36 | 71.15 |
| Top | 90.17 | 94.97 | 80.24 |
| IC50 | 2.221 ˟ 10^-3^ | 4.116 ˟ 10^-3^ | 1.282 ˟ 10^-3^ |
| logIC50 | -2.653 | -2.386 | -2.892 |
| Span | 10.03 | 9.609 | 9.096 |
| **95% CI (profile likelihood)** |  |  |  |
| Bottom | 79.57 to 80.67 | 80.85 to 88.28 | 68.00 to 73.04 |
| Top | 89.64 to 90.75 | 92.98 to 98.25 | 77.54 to 84.50 |
| IC50 | 1.650 ˟ 10^-3^ | 6.257 ˟ 10^-4^ to 1.921 ˟ 10^-2^ | 2.668 ˟ 10^-4^ to 8.219 ˟ 10^-3^ |
| logIC50 | -2.738 to -2.524 | -3.204 to -1.716 | -3.574 to -2.085 |
| **Goodness of Fit** |  |  |  |
| Degrees of Freedom | 3 | 3 | 3 |
| R squared | 9.989 ˟ 10^-1^ | 9.706 ˟ 10^-1^ | 9.719 ˟ 10^-1^ |
| Sum of Squares | 7.952 ˟ 10^-2^ | 1.841 | 1.590 |
| Sy.x | 1.628 ˟ 10^-1^ | 7.834 ˟ 10^-1^ | 7.280 ˟ 10^-1^ |
| **Constraints** |  |  |  |
| IC50 | IC50 > 0 | IC50 > 0 | IC50 > 0 |
| **Number of points** |  |  |  |
| # of X values | 6 | 6 | 6 |
| # Y values analyzed | 6 | 6 | 6 |

**Table S3.** The IC50 results of HEK-293 48h

| (Inhibitor) vs. response (three parameters) | rGO/MIL125(Ti)@PdL@Dox | rGO/MIL125(Ti)@PdL | rGO/MIL125(Ti)@Dox |
| --- | --- | --- | --- |
| **Best-fit values** |  |  |  |
| Bottom | 75.21 | 81.74 | 52.95 |
| Top | 78.66 | 94.94 | 74.22 |
| IC50 | 5.175 ˟ 10^-4^ | 2.050 ˟ 10^-3^ | 1.281 ˟ 10^-2^ |
| logIC50 | -3.286 | -2.688 | -1.892 |
| Span | 3.455 | 13.20 | 21.28 |
| **95% CI (profile likelihood)** |  |  |  |
| Bottom | 73.81 to 76.17 | 80.43 to 82.86 | -infinity to ??? |
| Top | 76.77 to ??? | 93.74 to 96.32 | 66.94 to ??? |
| IC50 | ??? to 1.117 ˟ 10^-2^ | 1.219 ˟ 10^-3^ to 3.474 ˟ 10^-3^ | ??? to +infinity |
| logIC50 | ??? to -1.952 | -2.914 to -2.459 | ??? to +infinity |
| **Goodness of Fit** |  |  |  |
| Degrees of Freedom | 3 | 3 | 3 |
| R squared | 9.043 ˟ 10^-1^ | 9.969 ˟ 10^-1^ | 8.450 ˟ 10^-1^ |
| Sum of Squares | 6.617 ˟ 10^-1^ | 3.740 ˟ 10^-1^ | 38.79 |
| Sy.x | 4.697 ˟ 10^-1^ | 3.531 ˟ 10^-1^ | 3.596 |
| **Constraints** |  |  |  |
| IC50 | IC50 > 0 | IC50 > 0 | IC50 > 0 |
| **Number of points** |  |  |  |
| # of X values | 6 | 6 | 6 |
| # Y values analyzed | 6 | 6 | 6 |

**Table S4.** The IC50 results of HT-29 24h

| (Inhibitor) vs. response (three parameters) | rGO/MIL125(Ti)@PdL@Dox | rGO/MIL125(Ti)@PdL | rGO/MIL125(Ti)@Dox |
| --- | --- | --- | --- |
| **Best-fit values** |  |  |  |
| Bottom | 75.63 | 86.74 | 60.58 |
| Top | 78.43 | 91.66 | 65.13 |
| IC50 | 4.403 ˟ 10^-4^ | 3.428 ˟ 10^-3^ | 3.332 ˟ 10^-3^ |
| logIC50 | -3.356 | -2.465 | -2.477 |
| Span | 2.793 | 4.923 | 4.545 |
| **95% CI (profile likelihood)** |  |  |  |
| Bottom | 75.45 to 75.80 | -4453 to 89.16 | 32.14 to 62.81 |
| Top | 77.98 to 79.02 | 89.79 to ??? | 63.38 to ??? |
| IC50 | 2.451 ˟ 10^-4^ to 7.592 ˟ 10^-4^ | ??? to 60.71 | ??? to 4.087 ˟ 10^-1^ |
| logIC50 | -3.611 to -3.120 | ??? to 1.783 | ??? to -3.886 ˟ 10^-1^ |
| **Goodness of Fit** |  |  |  |
| Degrees of Freedom | 3 | 3 | 3 |
| R squared | 9.952 ˟ 10^-1^ | 9.048 ˟ 10^-1^ | 9.016 ˟ 10^-1^ |
| Sum of Squares | 1.851 ˟ 10^-2^ | 1.723 | 1.528 |
| Sy.x | 7.855 ˟ 10^-1^ | 7.578 ˟ 10^-1^ | 7.136 ˟ 10^-1^ |
| **Constraints** |  |  |  |
| IC50 | IC50 > 0 | IC50 > 0 | IC50 > 0 |
| **Number of points** |  |  |  |
| # of X values | 6 | 6 | 6 |
| # Y values analyzed | 6 | 6 | 6 |

**Table S5.** The IC50 results of HT-29 48h

| (Inhibitor) vs. response (three parameters) | rGO/MIL125(Ti)@PdL@Dox | rGO/MIL125(Ti)@PdL | rGO/MIL125(Ti)@Dox |
| --- | --- | --- | --- |
| **Best-fit values** |  |  |  |
| Bottom | 65.51 | 83.19 | 52.05 |
| Top | 79.43 | 90.29 | 62.20 |
| IC50 | 3.648 ˟ 10^-4^ | 1.293 ˟ 10^-3^ | 1.652 ˟ 10^-4^ |
| logIC50 | -3.438 | -2.888 | -3.782 |
| Span | 13.92 | 7.098 | 10.15 |
| **95% CI (profile likelihood)** |  |  |  |
| Bottom | 62.79 to 67.92 | 79.90 to 84.95 | 50.84 to ??? |
| Top | 73.41 to ??? | 87.82 to 94.52 | 57.43 to ??? |
| IC50 | ??? to 2.329 ˟ 10^-3^ | 1.966 ˟ 10^-4^ to 1.266 ˟ 10^-2^ | ??? to 8.925 ˟ 10^-4^ |
| logIC50 | ??? to -2.633 | -3.706 to -1.898 | ??? to -3.049 |
| **Goodness of Fit** |  |  |  |
| Degrees of Freedom | 3 | 3 | 3 |
| R squared | 9.550 ˟ 10^-1^ | 9.581 ˟ 10^-1^ | 9.692 ˟ 10^-1^ |
| Sum of Squares | 4.139 | 1.465 | 9.246 ˟ 10^-1^ |
| Sy.x | 1.175 | 6.988 ˟ 10^-1^ | 5.552 ˟ 10^-1^ |
| **Constraints** |  |  |  |
| IC50 | IC50 > 0 | IC50 > 0 | IC50 > 0 |
| **Number of points** |  |  |  |
| # of X values | 6 | 6 | 6 |
| # Y values analyzed | 6 | 6 | 6 |

**
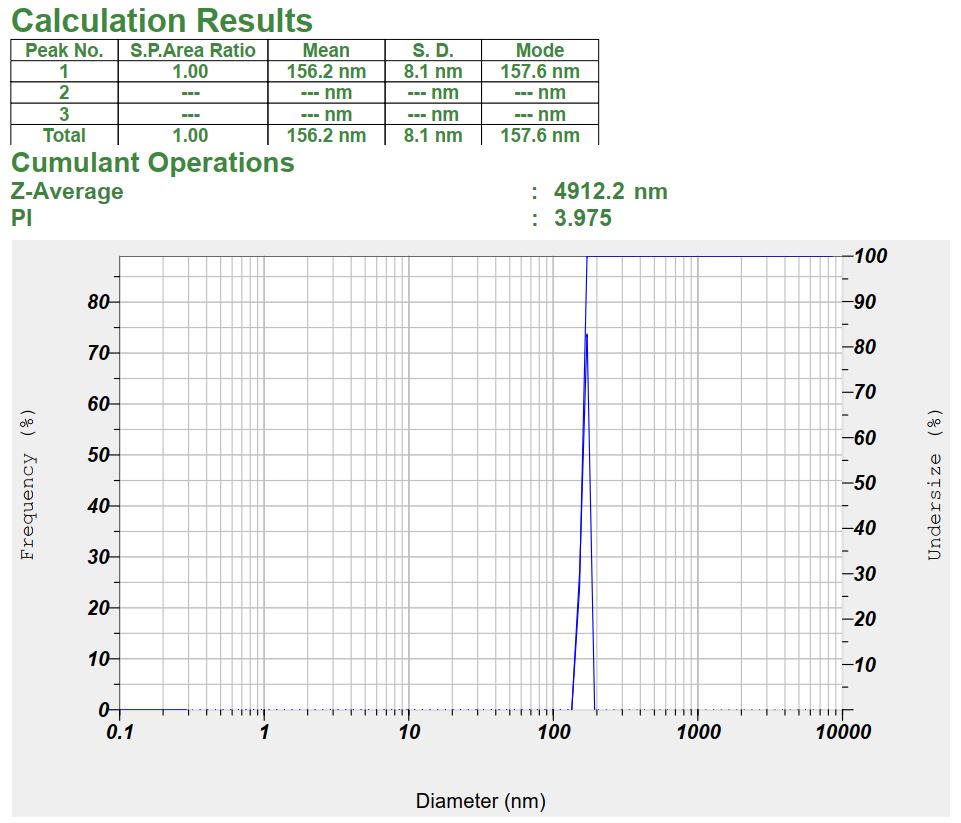
**

**Figure S3.** The DLS result of prepared nanocomposite in DI water.


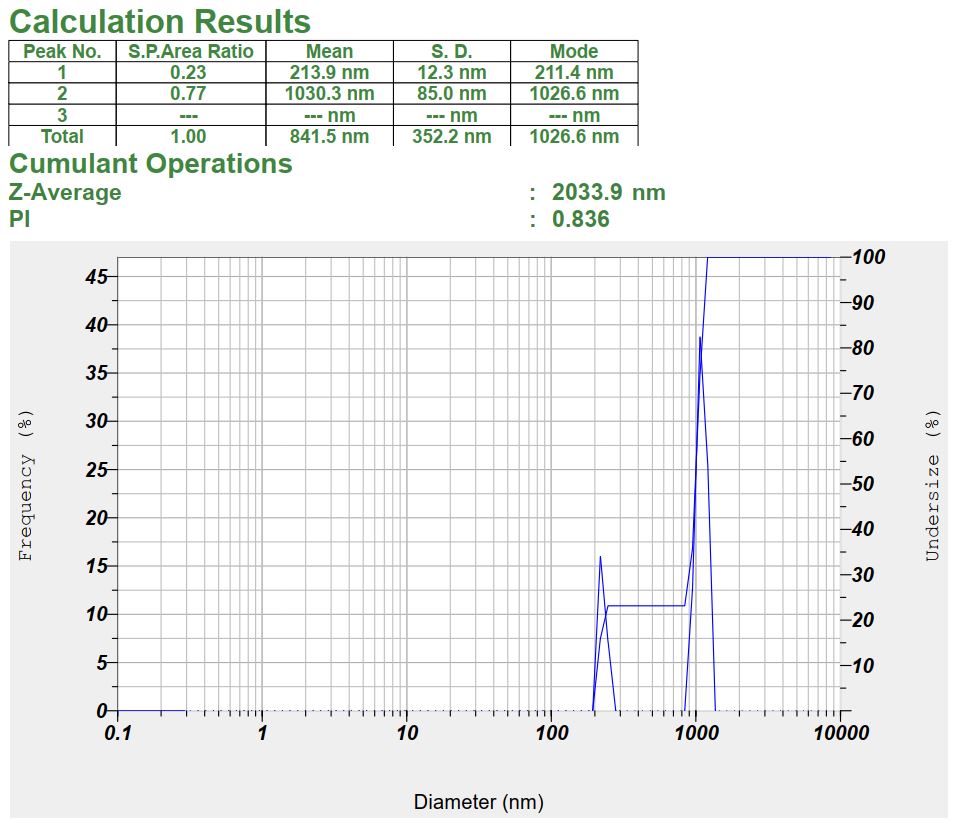


**Figure S4.** The DLS result of prepared nanocomposite in PBS. (t=0 h)


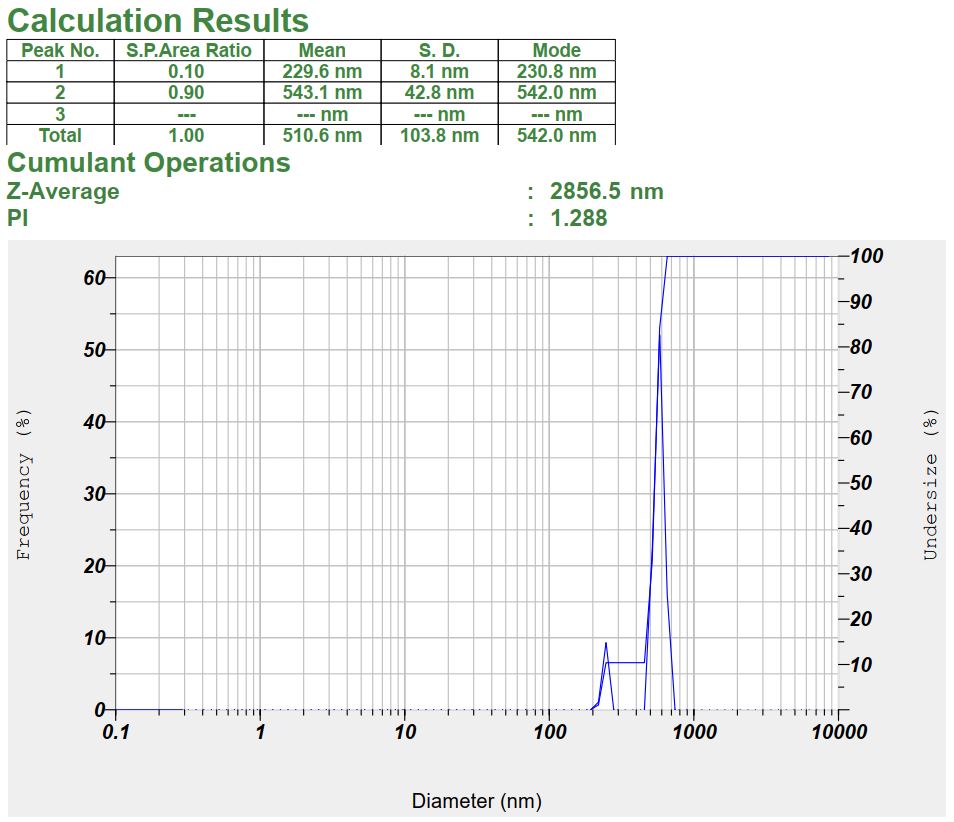


**Figure S5.** The DLS result of prepared nanocomposite in PBS. (t=12 h)


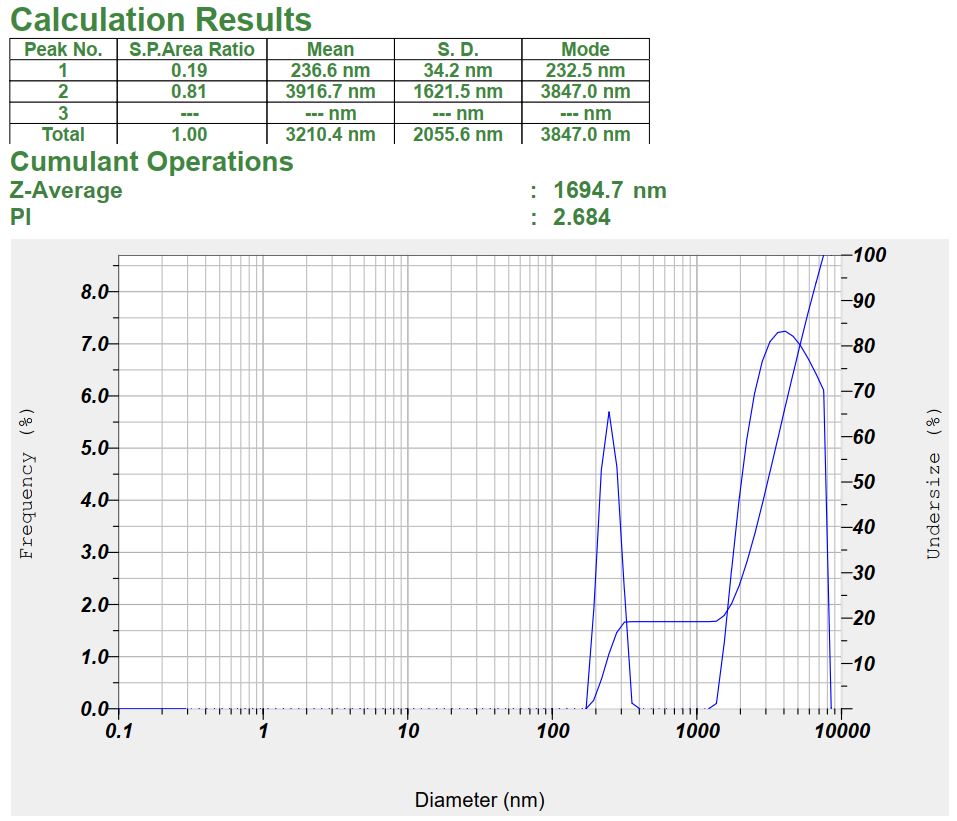


**Figure S6.** The DLS result of prepared nanocomposite in PBS. (t=24 h)


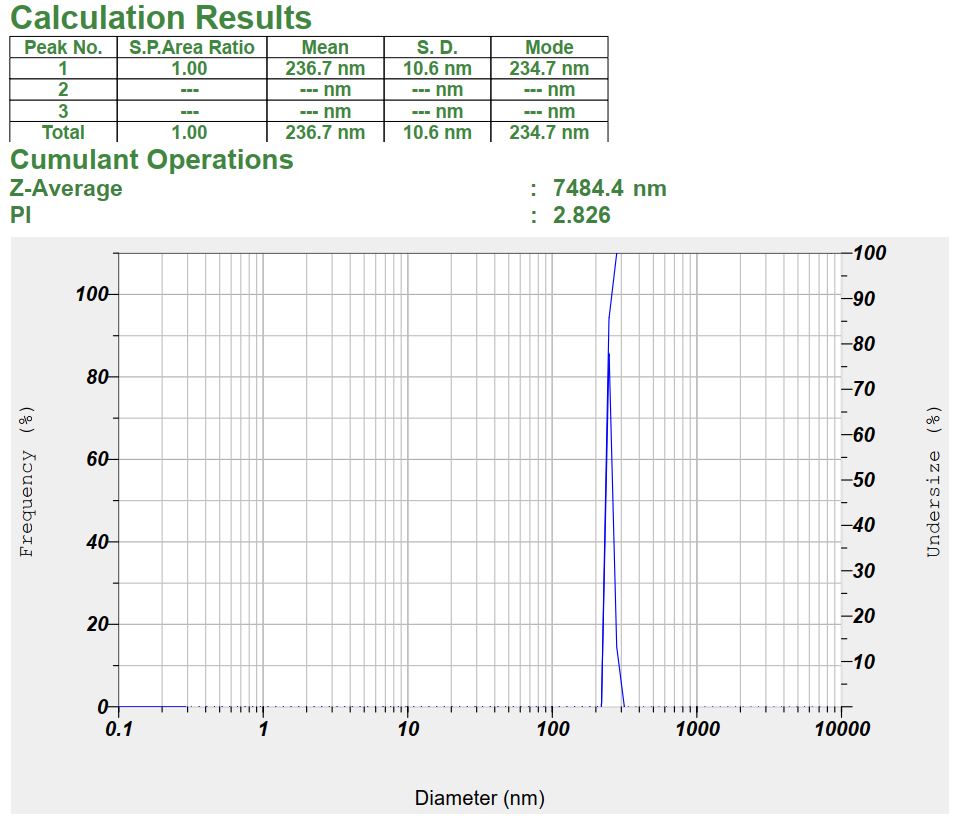


**Figure S7.** The DLS result of prepared nanocomposite in PBS. (t=36 h)


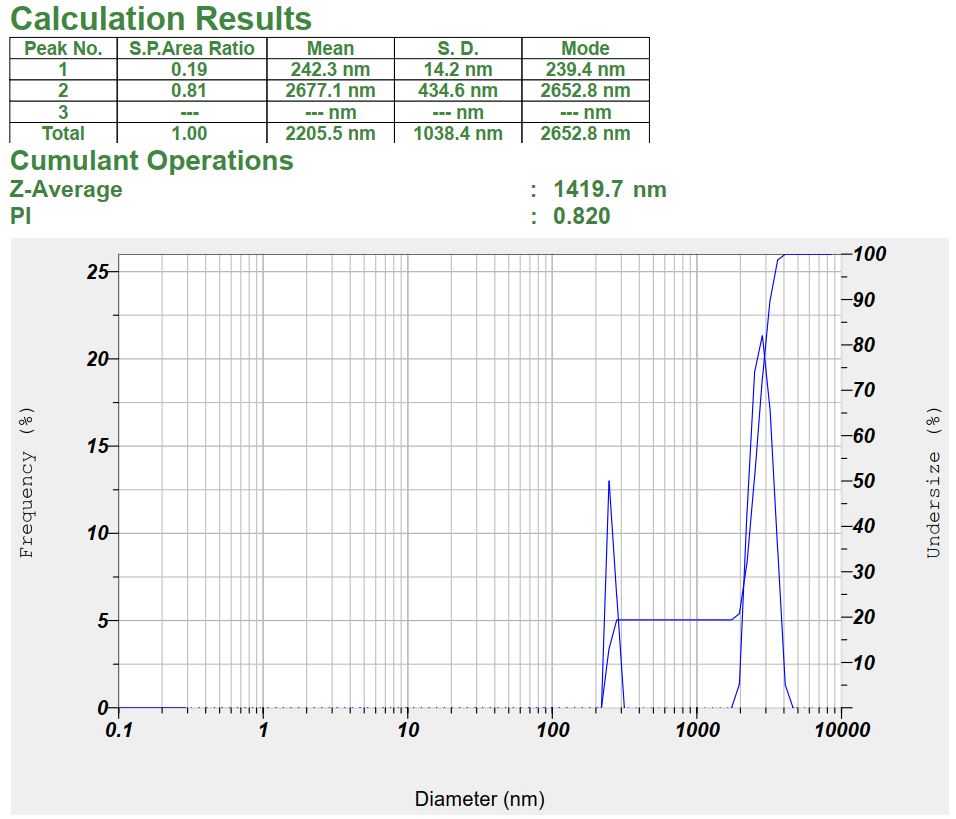


**Figure S8.** The DLS result of prepared nanocomposite in PBS. (t=48 h)


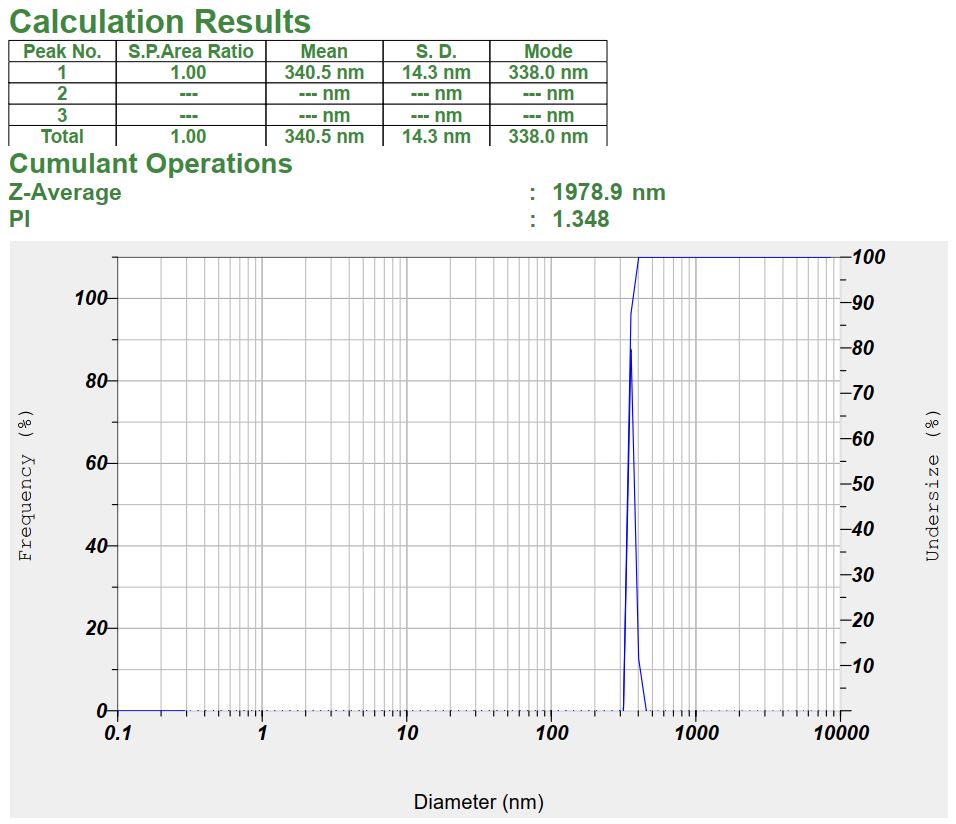


**Figure S9.** The DLS result of prepared nanocomposite in DMEM+10% FBS. (t=0 h)

**
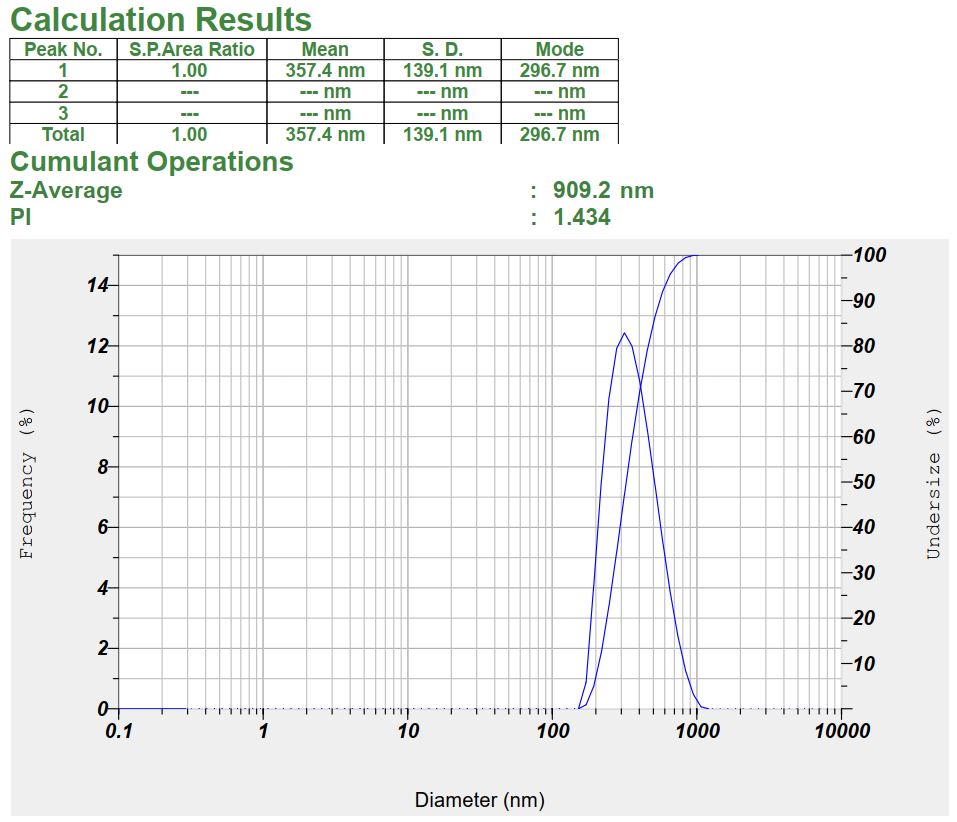
**

**Figure S10.** The DLS result of prepared nanocomposite in DMEM+10% FBS. (t=12 h)


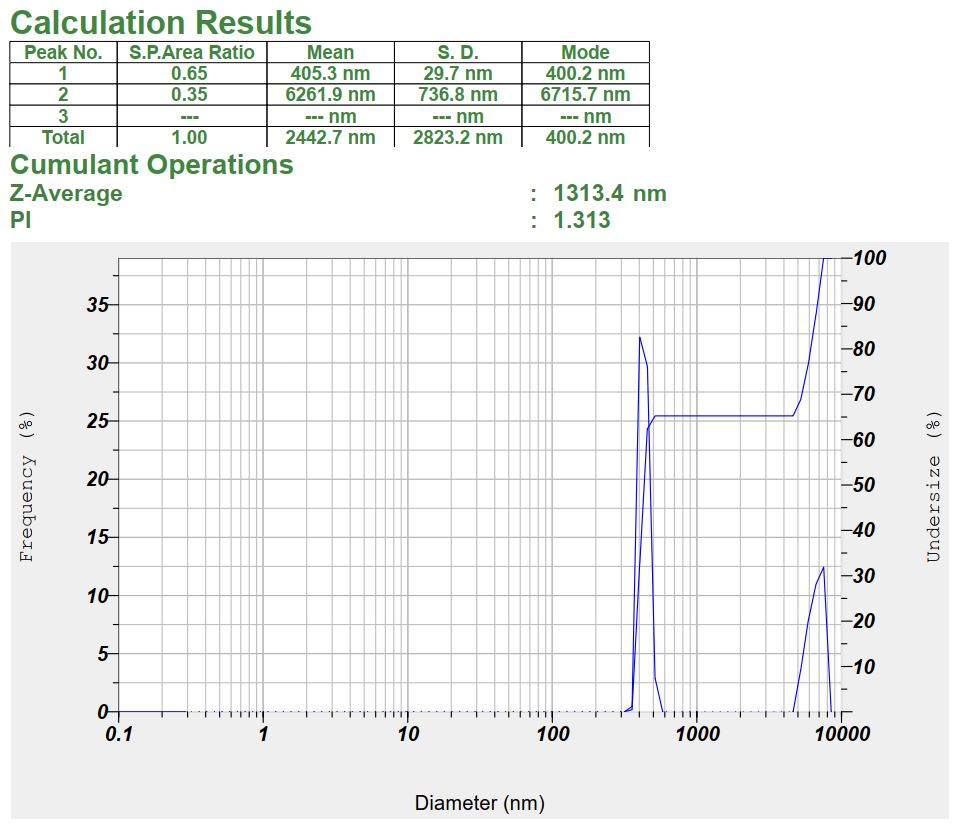


**Figure S11.** The DLS result of prepared nanocomposite in DMEM+10% FBS. (t=24 h)


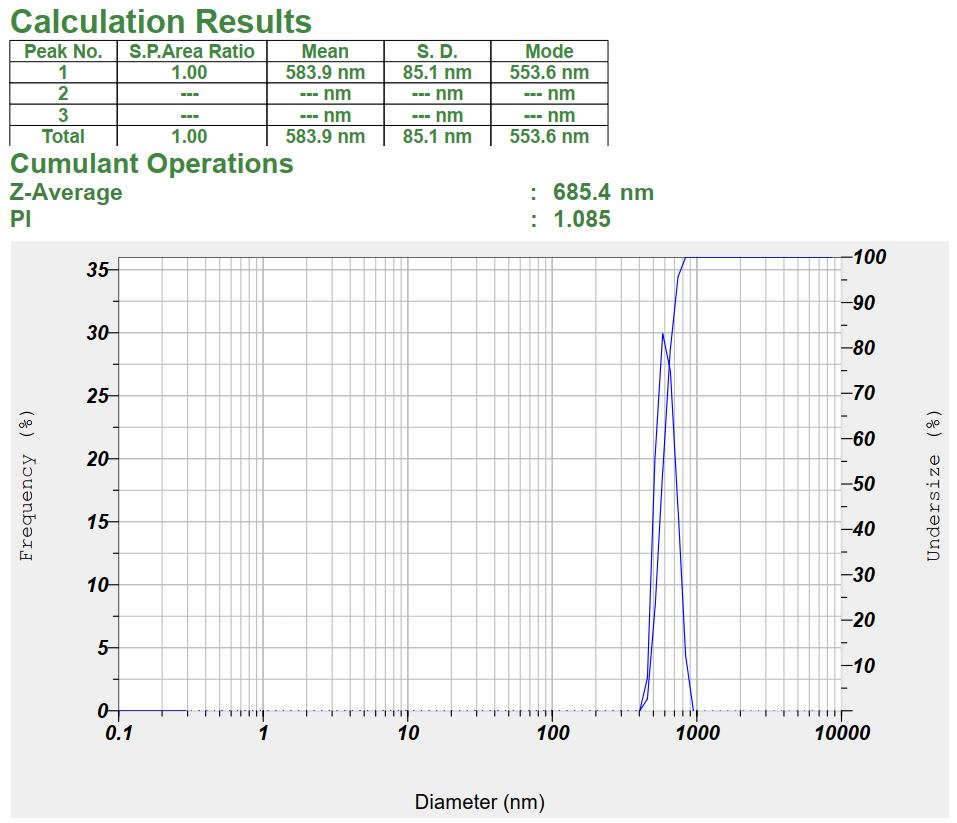


**Figure S12.** The DLS result of prepared nanocomposite in DMEM+10% FBS. (t=36 h)


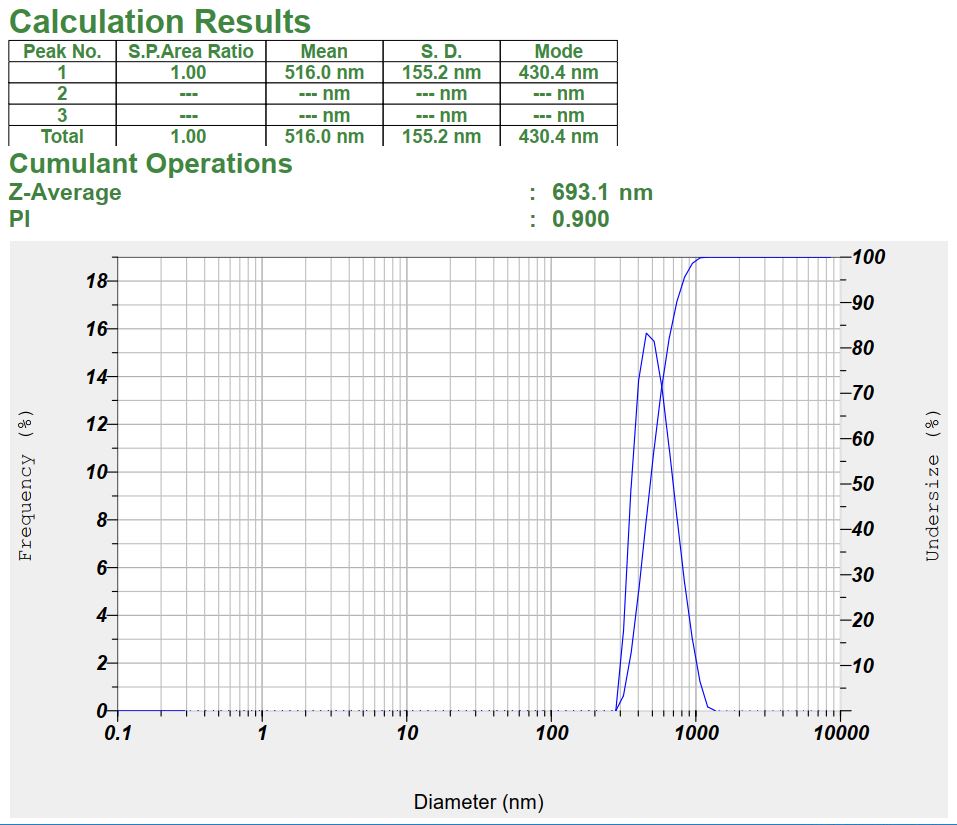


**Figure S13.** The DLS result of prepared nanocomposite in DMEM+10% FBS. (t=48 h)


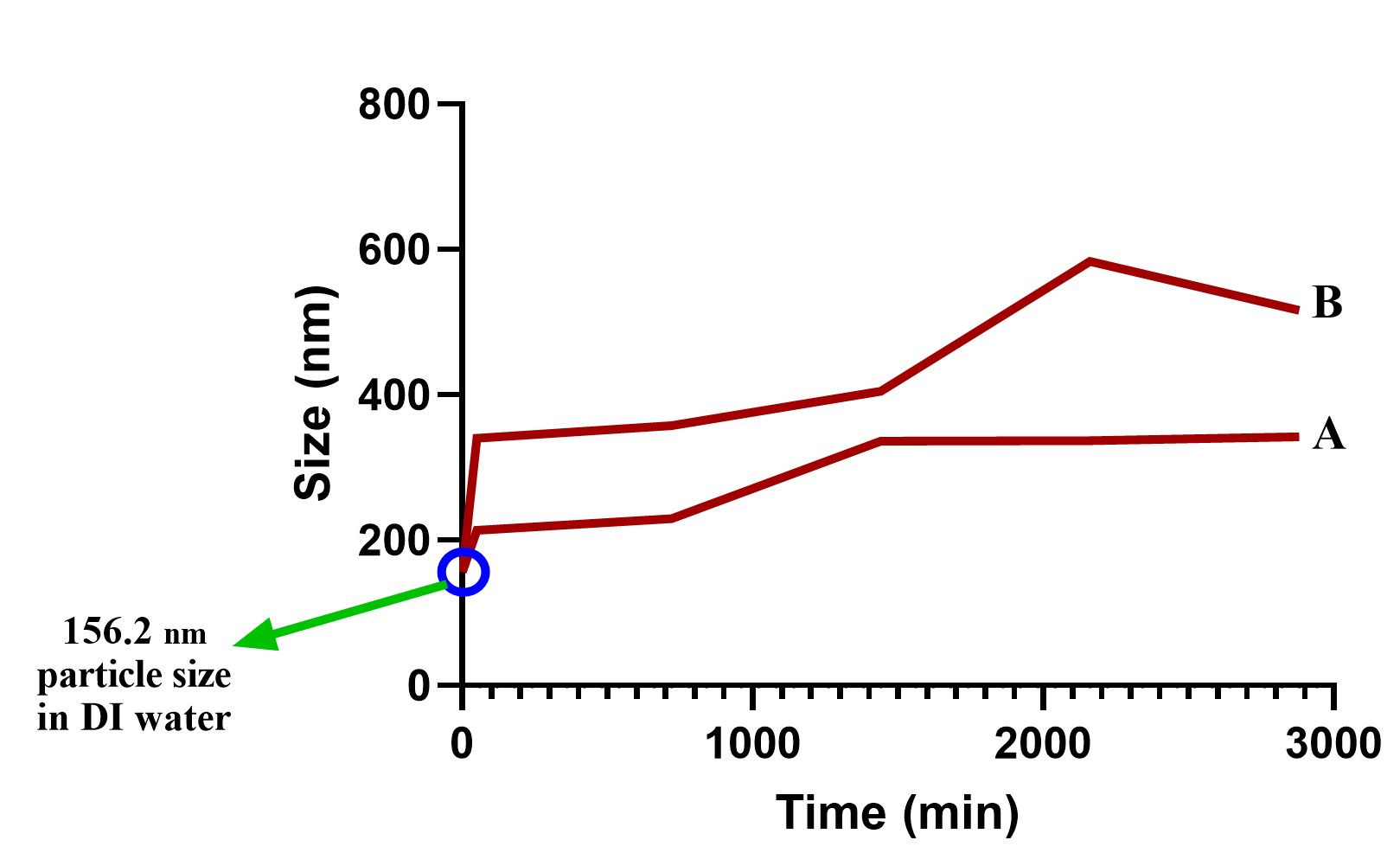


**Figure S14**. The biological stability of prepared nanocomposite in **A**: PBS and **B**: DMEM+10% FBS.

**
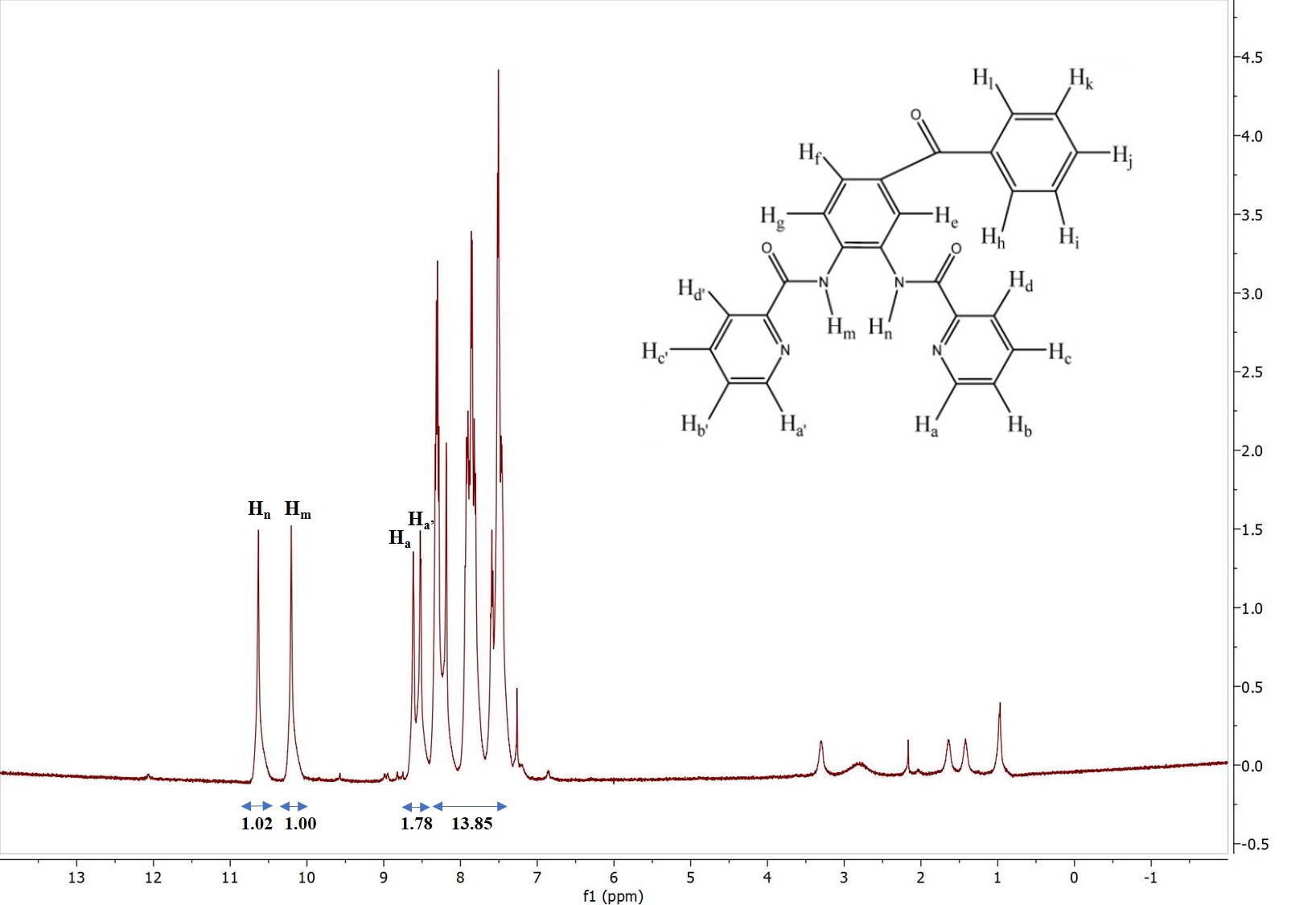
**

**Figure S15.** The labeled ^1^HNMR spectra of L.


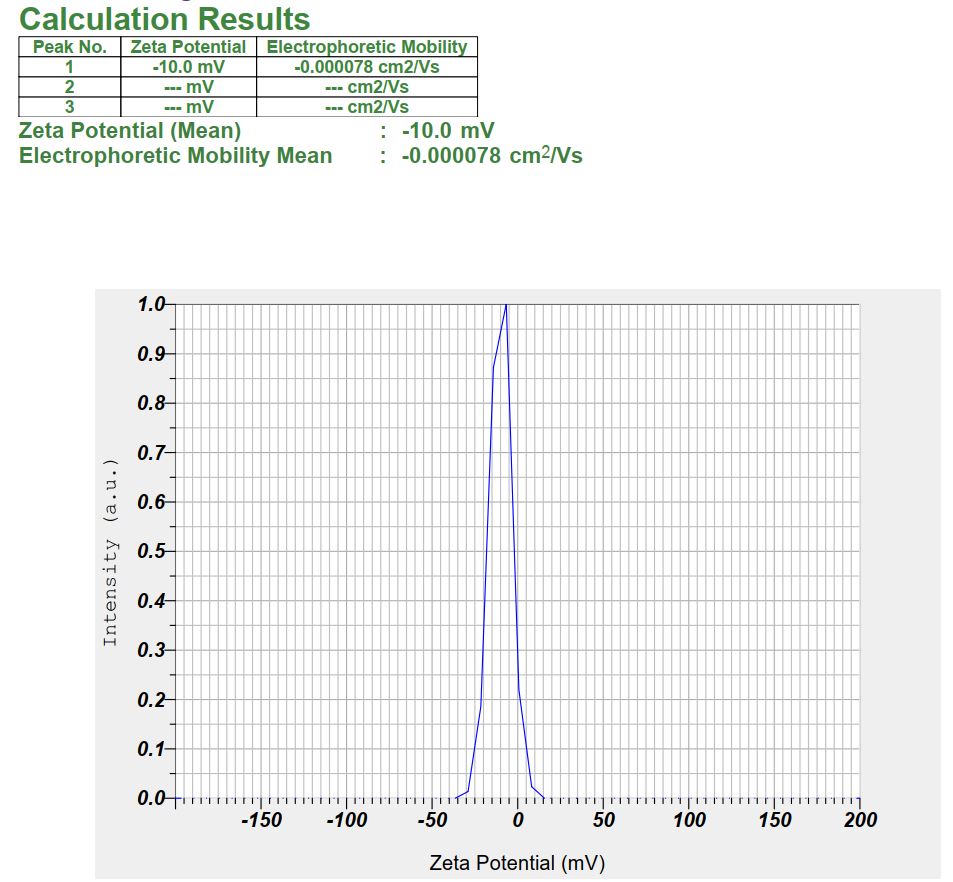


**Figure S16.** The Zeta potential of prepared nanocomposite.
